# Supplementary material for: Identification of Freezing-Responsive microRNAs and Their Targets in Chinese Jujube by Small RNA and Degradome Sequencing
Source: Int J Mol Sci. 2026 Sep 4;27(17):7912. doi: 10.3390/ijms27177912 (PMC13566890; doi:10.3390/ijms27177912)
Supplement: Supplementary file 1 [file ijms-27-07912-s001.zip › Supplementary Material S2/novel_miR_167.pdf]

Provisional ID	:	novel_miR_167
Score total	:	103516.5
Score for star read(s)	:	3.9
Score for read counts	:	103510.4
Score for mfe	:	0.5
Score for randfold	:	1.6
Score for cons. seed	:	
Total read count	:	203043
Mature read count	:	179050
Loop read count	:	24
Star read count	:	23969

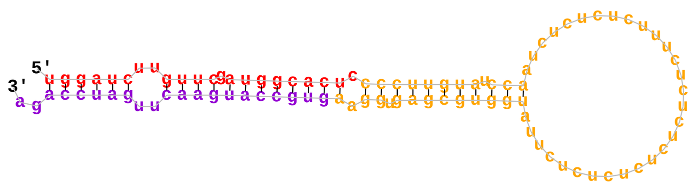

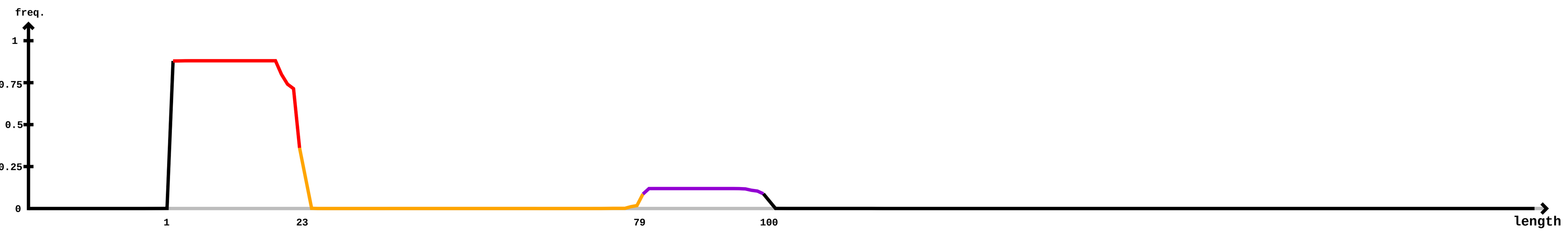

Mature **Star**

```
5'- uccaucuuguucguucacccucuggaucuuguucgauggcacuccccuuguauccaaucucucucuuucucucucucucucucuuauggugcgaguggaagugccaugaacuugauccagaaggugcucucgaacaaguuacaccuacuuaauaucuacuuucuucuuucuccauuacuauuauuaaugaugguuucuuuuuucugauucugauuuuuaauuuauuuauuuguuuuuuguguuuguguuu -3' obs
    uccaucuuguucguucacccucuggaucuuguucgauggcacuccccuuguauccaaucucucucuuucucucucucucucucuuauggugcgaguggaagugccaugaacuugauccagaaggugcucucgaacaaguuacaccuacuuaauaucuacuuucuucuuucuccauuacuauuauuaaugaugguuucuuuuuucugauucugauuuuuaauuuauuuauuuguuuuuuguguuuguguuu     exp
    .....((((((((..((((.((((((((..((((.((((((((.((((((((.(((.........................))))))))).)).))))))))))))..)))))))).))))....))))))))..((((..((.(((..................((((((.((....)).))))))..((........))............................))).))..))))..   reads  mm  sample
    ...aucuuguucguucacccucugg......     2  0  LT-1
    ......uuguucguucacccucuggaucuuguuc......     1  0  LT-1
    .............uucacccucuggaucuuguu......     2  0  LT-2
    ..................ccucuggaucuuguucgauggc......     1  0  JCK-2
    ..................ccucuggaucuuguucgauggc......     1  0  LT-3
    ..................ccucuggCucuuguucgauggc......     1  1  LT-1
    ..................ccucuggaucuuguucgauggc......     1  0  JCK-1
    ..................ccucuggaucuuguucgauggc......     2  0  LT-2
    ..................ccucuggaucuuguucgauggc......     1  0  LT-1
    ..................ccucuggaucuuguucgauggcacA......     1  1  JCK-3
    ...................cucuggaucuuguuUgau......     2  1  JCK-3
    ...................cucuggaucuuguucgCuggc......     2  1  LT-3
    ...................cucuggaucuuguucgauggc......     1  0  JCK-3
    ...................cucuggaucuuguucgauggc......     1  0  LT-1
    ...................cucuggaucuuguucgauggc......     9  0  LT-3
    ...................cucuggaucuuguucgauggc......     2  0  JCK-1
    ...................cucuggaucuuguucgauggc......     3  0  JCK-2
    ...................Aucuggaucuuguucgauggcacu......     1  1  JCK-1
    ....................ucuggaucuuguucgauggc......     3  0  JCK-1
    ....................ucuggaucuuguucgauggc......     1  0  JCK-2
    ....................ucuggaucuuguucgauAgca......     1  1  JCK-3
    ....................ucuggaucuuguucgauggca......     1  0  JCK-1
    ....................ucuggaucuuguucgauggca......     4  0  LT-3
    ....................ucuggaucuuguucgauggca......     2  0  LT-2
    ....................ucuggaucuuguucgauggca......     1  0  JCK-3
    ....................ucuggaucuuguucgauggcac......     1  0  JCK-1
    ....................ucuggaucuuguucgauggcac......     1  0  JCK-2
    ....................ucuggaucuuguucgauggcac......     1  0  LT-1
    ....................ucuggaucuuguucgauggcacuc......     1  0  LT-3
    ....................ucuggaucuuguucgauggcacuc......     2  0  JCK-3
    ....................ucuggaucuuguucgauggcacuc......     1  0  JCK-1
    ....................ucuggaucuuguucgauggcacuc......     3  0  JCK-2
    .....................cuggaucuuguucgauggc......     1  0  JCK-1
```

```
                      Mature                                                                                  Star
uccaucuuguucguucacccucuggaucuuguucgauggcacuccccuuguauccaaucucucucuuucucucucucucucuuauggugcgaguggaagugccaugaacuugauccagaaggugcucucgaacaaguuacaccuacuuaauaucuacuuucuucuuucuccauuacuauuauuaaugaugguuucuuuuuucugauucugauuuuuaauuuauuuauuuguuuuuguguuuguguuu
.....................cuggaucuuguucgauggc........    2     0    LT-1
.....................cuggaucuuguucgauggc........    3     0    LT-2
.....................cuggaucuuguucgauggca........   2     0    LT-1
.....................cuggaucuuguucgauggcac........  2     0    LCK-3
.....................cuggaucuuguucgauggcacu........ 2     0    LT-1
.....................cuggaucuuguucgauggcacu........ 8     0    JCK-3
.....................cuggaucuuguucgauggcacu........ 6     0    JCK-1
.....................cuggaucuuguucgauggcacu........ 7     0    LT-3
.....................cuggaucuuguucgauggcacu........ 5     0    LT-2
.....................cuggaucuuguucgauggcacu........ 16    0    JCK-2
.....................cuggaucuuguucgauggcacuU........ 1    1    LT-3
.....................cuggaucuuguucgauggcacuc........ 2    0    JCK-3
.....................cuggaucuuguucgauggcacuc........ 1    0    LT-3
.....................cuggaucuuguucgauggcacuc........ 3    0    LT-2
.....................cuggaucuuguucgauggcacuc........ 3    0    JCK-1
.....................cuggaucuuguucgauggcacuc........ 2    0    LT-1
.....................cuggaucuuguucgauggcacuc........ 4    0    JCK-2
......................uggaucuuguuNgauggc........    2     1    JCK-1
......................uggaucuuguucgCuggc........    7     1    LT-1
......................uggaucCuguucgauggc........    1     1    LCK-3
......................uggaucuuguucAauggc........    1     1    LCK-3
......................uggaucuuguucgGuggc........    1     1    LCK-1
......................uAgaucuuguucgauggc........    1     1    LCK-2
......................ugNaucuuguucgauggc........    13    1    LT-2
......................uggaucuuguucgaNggc........    6     1    LCK-1
......................uggaucuuguucgauggc........    186   0    JT-1
......................uggaucuuguucgaugUc........    1     1    LT-2
......................uggaucuuguuUgauggc........    1     1    LT-1
......................ugAaucuuguucgauggc........    1     1    LT-3
......................uNgaucuuguucgauggc........    1     1    JCK-2
......................uggaucuuguucgauAgc........    1     1    LT-2
......................ugAaucuuguucgauggc........    1     1    LT-2
......................uAgaucuuguucgauggc........    1     1    LT-1
......................uggaucuuguucgCuggc........    8     1    LCK-3
......................uggaucuuguucgaugAc........    4     1    LCK-3
......................uggaucuuguucgCuggc........    1     1    LCK-2
......................ugAaucuuguucgauggc........    1     1    LT-1
......................uggaucuuguucgauggc........    253   0    JCK-1
......................uggaucuuguucgCuggc........    19    1    LT-2
......................uggaucuuguucgauggN........    1     1    JCK-3
......................uggauAuuguucgauggc........    1     1    LT-1
......................uggaucuuAuucgauggc........    1     1    LCK-3
......................uggaucuuguucgGuggc........    1     1    LT-1
......................uggaucuuguucgauggU........    26    1    LCK-3
......................uggaucuuguucgauggc........    182   0    JT-3
......................uggaucuuguucgCuggc........    5     1    LT-3
......................uggaucuuguucgauggc........    134   0    JT-2
......................ugAaucuuguucgauggc........    1     1    LCK-3
......................uggaucuuguucgaugCc........    1     1    LCK-3
......................uggaucuuguucgauggc........    489   0    LCK-2
......................uggaucuuguucgCuggc........    1     1    JT-2
......................ugCaucuuguucgauggc........    2     1    LT-2
......................uggaucuuguucgNuggc........    2     1    LCK-1
......................uggCucuuguucgauggc........    1     1    JCK-2
......................uggaucuuguucgauggU........    23    1    LCK-1
......................uggaucuugCucgauggc........    2     1    LT-2
......................uggaucuuguucgauggU........    1     1    JCK-3
......................uggaCcuuguucgauggc........    1     1    LT-2
......................uUgaucuuguucgauggc........    2     1    LT-2
......................uAgaucuuguucgauggc........    1     1    LT-2
......................uggCucuuguucgauggc........    2     1    LT-3
......................uggaucuuguucgauggU........    7     1    LT-3
......................uggaucuuguucgauggA........    2     1    LCK-3
......................uggaucuuguucgauCgc........    1     1    LCK-3
......................uggaucuuguucgaCggc........    1     1    JCK-2
......................uggaucuuguucgaugAc........    3     1    LT-2
......................uNgaucuuguucgauggc........    1     1    JCK-3
......................uggaucuuguucUauggc........    3     1    LT-2
......................uggaucuugCucgauggc........    1     1    LT-3
......................uggaucuuguucgauggc........    1716  0    LT-3
```

Mature **Star**

```
uccaucuuguucguucacccucuggaucuuguucgauggcacucccuuguauccaaucucucucuuucucucucucucucuuauggugcgaguggaagugccaugaacuugauccagaaggugcucucgaacaaguuacaccuacuuaauaucuacuuucuucuuucuccauuacuauuauuaaugaugguuucuuuuucugauucugauuuuuaauuuauuuauuuguuuuuguguuuguguuu
......................uggaucuuguucgauggc      377    0    JCK-2
......................uggaucuuguucgauggA      2      1    JCK-3
......................uggCucuuguucgauggc      37     1    LT-2
......................uggaucuuguucgauggc      522    0    LCK-1
......................uggaucuuguucgauggU      5      1    LT-1
......................uggaucuuguCcgauggc      3      1    JCK-2
......................uggaucuuguucgauAgc      1      1    LCK-3
......................uggaucuugCucgauggc      1      1    LT-1
......................uggGucuuguucgauggc      1      1    LCK-3
......................uggaucuuguucgCuggc      2      1    LCK-1
......................uggauUuuguucgauggc      1      1    LT-2
......................uggaucCuguucgauggc      1      1    LT-2
......................uggaucuuguucgauggc      4850   0    LT-2
......................Cggaucuuguucgauggc      1      1    LCK-3
......................uggaucuuguucgauggA      6      1    LT-3
......................uggCucuuguucgauggc      2      1    LT-1
......................uggaucuugCucgauggc      1      1    JCK-2
......................ugAaucuuguucgauggc      1      1    LCK-2
......................uggaucuuguucUauggc      1      1    LT-1
......................uggaucuuguucgauggU      3      1    LT-2
......................uAgaucuuguucgauggc      5      1    LCK-3
......................uggCucuuguucgauggc      9      1    LCK-3
......................uggaucuuguucgauggU      22     1    LCK-2
......................uggaucuuguucgCuggc      1      1    JT-3
......................uggaucuuguucgauggc      261    0    JCK-3
......................uggaucuuguucgauAgc      1      1    JCK-1
......................uggaucuuguucgauggc      2116   0    LT-1
......................uggaucuuguucgauggA      2      1    LT-2
......................uggaucuuguucgauggG      2      1    LT-3
......................uggaucuuguucAauggc      1      1    LT-2
......................uggGucuuguucgauggc      1      1    LT-1
......................uggaucuuguucgauggc      5148   0    LCK-3
......................uAgaucuuguucgauggc      2      1    LT-3
......................uggCucuuguucgauggca     2      1    LT-3
......................uggaucuuguucgCuggca     1      1    LT-3
......................uggaucuuUuucgauggca     1      1    LT-2
......................uggaucuuguucgauggca     377    0    JT-1
......................uggaucuuguucUauggca     1      1    LT-3
......................uggaucuuguucgauAgca     4      1    JCK-1
......................uggaucuuAuucgauggca     1      1    LCK-2
......................uggaucuuguucgauggcU     21     1    LT-2
......................uggCucuuguucgauggca     7      1    LCK-2
......................uggaucuuguucgauAgca     4      1    JCK-2
......................uggaucuuguucUauggca     3      1    LT-2
......................uggCucuuguucgauggca     3      1    LCK-1
......................uggCucuuguucgauggca     2      1    JCK-1
......................uggaucuuguucgauggcG     1      1    LT-3
......................uggaucuuguucgauAgca     2      1    LCK-2
......................uggaucuuguucgGuggca     1      1    LT-3
......................uggaucuuguucgauggcU     11     1    LT-1
......................uggaucuuguucgauggUa     1      1    LCK-3
......................uggaucuuguucgaCggca     1      1    JT-1
......................uggaucuuguucgauggcC     1      1    JT-1
......................uggaucuuguucgauggca     281    0    JT-3
......................uggaucuuguucgaugAca     2      1    LT-1
......................uggCucuuguucgauggca     1      1    LT-1
......................uggaucuuguuAgauggca     1      1    LCK-2
......................uggNucuuguucgauggca     1      1    JCK-2
......................uggaucuuguucgauggcU     1      1    JCK-3
......................uggaucuuguucgauAgca     1      1    LT-2
......................uggaucuuguucgauggcU     2      1    JT-2
......................uggaucuuguucgauggcG     1      1    JCK-1
......................uAgaucuuguucgauggca     1      1    LCK-3
......................uggaucuuguucgauggcU     24     1    LCK-1
......................ugNaucuuguucgauggca     1      1    LT-2
......................uggaucuuguucNauggca     1      1    LCK-1
......................uggaucuuguucgCuggca     10     1    LT-2
......................uggaucuuguucgauggcG     2      1    JT-3
......................uggaucuuguucgauAgca     1      1    JCK-3
......................uggaucuuguucgauggca     1539   0    LCK-1
```

Mature **Star**

```
uccaucuuguucguucacccucuggaucuuguucgauggcacuccccuuguauccaaucucucucuuucucucucucucucuuauggugcgaguggaagugccaugaacuugauccagaaggugcucucgaacaaguuacaccuacuuaauaucuacuuucuucuuucuccauuacuauuauuaaugaugguuucuuuuuucugauucugauuuuuaauuuauuuauuuguuuuuguguuuguguuu
......................uggaucuuguGcgauggca        1     1   JCK-1
......................uggaucuuguucgauggca        259   0   JCK-1
......................uggaucuuguucgCuggca        3     1   LCK-2
......................uggaucuuguucgauggcC        1     1   LT-1
......................uggaucuuguucgauggca        2503  0   LCK-3
......................ugAaucuuguucgauggca        1     1   LT-2
......................uggaucuuguucAauggca        1     1   LCK-1
......................uggCucuuguucgauggca        7     1   LCK-3
......................uggCucuuguucgauggca        10    1   LT-2
......................uggGucuuguucgauggca        1     1   LCK-3
......................uggaucuuAuucgauggca        1     1   LT-1
......................uggaucuuguucgauggca        1462  0   LCK-2
......................uggaucuuAuucgauggca        1     1   LT-2
......................uggaucuuguucgGuggca        1     1   LCK-1
......................uggaucuuguCcgauggca        1     1   LCK-3
......................ugAaucuuguucgauggca        1     1   LCK-2
......................uggGucuuguucgauggca        1     1   LCK-1
......................uggaucuuguucgauggcG        8     1   LCK-3
......................uggaucuuguucgauggcC        1     1   LT-3
......................uggaucuuguucgauggcC        2     1   LCK-3
......................uggaucuuguucgNuggca        7     1   LCK-1
......................uAgaucuuguucgauggca        1     1   LCK-2
......................uggaucuuAuucgauggca        1     1   LT-3
......................uggaucuuguucgauggca        411   0   JCK-2
......................uggaucuuguucgauggcU        24    1   LT-3
......................uggaucuCguucgauggca        1     1   LT-2
......................uggaucuuguucgaNggca        2     1   LCK-1
......................uggauNuuguucgauggca        2     1   LCK-1
......................ugAaucuuguucgauggca        1     1   LCK-3
......................uggaucuuguucUauggca        6     1   LCK-3
......................uggaucuuguucgauggcG        1     1   LT-1
......................uggaucuuguucgauggcC        1     1   LCK-1
......................uggaucuuguucgauggcU        8     1   JCK-2
......................uggaucuuguucgCuggca        1     1   JCK-1
......................uggaucuuguucgauggcU        13    1   LCK-3
......................uggaucuuguucgauggca        1101  0   LT-1
......................uggaucuuguucgauggcU        20    1   LCK-2
......................uggaucuuguucgaGggca        1     1   LCK-1
......................uAgaucuuguucgauggca        2     1   LT-3
......................uggaucuuguucgauggca        306   0   JCK-3
......................uggaucuuguuUgauggca        1     1   JT-1
......................ugAaucuuguucgauggca        2     1   LCK-1
......................uggaucuCguucgauggca        1     1   LCK-3
......................uggaucuuguucgauggca        1208  0   LT-3
......................uggaucuuguucgCuggca        2     1   LCK-3
......................Cggaucuuguucgauggca        1     1   JCK-2
......................uggaucuuguucgauggca        182   0   JT-2
......................uggaucuuguucgCuggca        2     1   LCK-1
......................uggaucuuguucgauggcC        5     1   LT-2
......................uggaucuuguucgCuggca        1     1   JCK-2
......................uAgaucuuguucgauggca        2     1   LT-2
......................uggaucuuguucgauggAa        2     1   LT-1
......................uggaucuuguucgauggca        1906  0   LT-2
......................uggaucuuguucgauggcU        7     1   JT-3
......................uggaucuuguucgauggcCc       1     1   LCK-3
......................uggaucuuguucgauggcac       704   0   LT-3
......................uggaucuuguucgauggcaU       26    1   LT-3
......................uggaucuuguucgauggcaU       13    1   LT-2
......................uggaucuuguucgauggcac       427   0   LCK-1
......................uggaucuuguuUgauggcac       1     1   LCK-3
......................uggaucuuguucgauggcCc       1     1   LT-1
......................uggaucuuguucgauggcac       108   0   JT-1
......................uggaucuuguucgauggcGc       1     1   LT-2
......................uggNucuuguucgauggcac       1     1   LCK-1
......................uggaucuuguucgauggcac       1673  0   LCK-3
......................uggaucuCguucgauggcac       1     1   LT-2
......................uggaucCuguucgauggcac       1     1   LT-3
......................uggaucuuguucgauAgcac       3     1   JCK-2
......................uggaucuuguucUauggcac       1     1   JT-1
......................uggaucuuguucgauAgcac       1     1   JCK-1
```

Mature **Star**

```
uccaucuuguucguucacccucuggaucuuguucgauggcacuccccuuguauccaaucucucucuuucucucucucucucuuauggugcgaguggaagugccaugaacuugauccagaaggugcucucgaacaaguuacaccuacuuaauaucuacuuucuucuuucuccauuacuauuauuaaugaugguuucuuuuuucugauucugauuuuuaauuuauuuauuuguuuuuguguuuguguuu
......................uggaucuuguucgauggcac......    427    0    LCK-2
......................uggaucuuguucgaugAcac......    1      1    LT-2
......................uggaucuuguucgaGggcac......    1      1    LCK-1
......................uggaucuuguucgauggcaU......    3      1    JT-3
......................uggaucuuguucgauggcac......    19     0    JT-2
......................uggaucuuguucgauggcaU......    3      1    JCK-1
......................uggaucuuguucgauAgcac......    3      1    JCK-3
......................uggaucuuguucgCuggcac......    1      1    LCK-3
......................uggaucuuguucgauggcaU......    12     1    LCK-3
......................uggCucuuguucgauggcac......    3      1    LT-2
......................uggaucuuguucgauggcUc......    6      1    LCK-3
......................uggaucuuguucgCuggcac......    1      1    LCK-1
......................uggaucuuguucgauggcUc......    1      1    LT-2
......................Cggaucuuguucgauggcac......    1      1    LT-3
......................uAgaucuuguucgauggcac......    1      1    LT-2
......................uggaucuuguucgauggcaU......    13     1    LT-1
......................uggCucuuguucgauggcac......    1      1    JCK-1
......................uggaucuuguucgauggcac......    571    0    LT-1
......................uggaucuuguucgCuggcac......    1      1    LT-1
......................uggaucuuguucgaCggcac......    1      1    LT-2
......................uggaucuuguucgCuggcac......    2      1    LT-3
......................uggaucuuguucgauggcac......    118    0    JCK-3
......................ugAaucuuguucgauggcac......    1      1    JCK-3
......................uAgaucuuguucgauggcac......    1      1    LCK-3
......................uggaCcuuguucgauggcac......    1      1    LT-2
......................uggaucuuguuUgauggcac......    1      1    LT-1
......................uggaucuuguucgauggcac......    40     0    JT-3
......................uggaucuuguucgauAgcac......    1      1    LT-2
......................ugNaucuuguucgauggcac......    4      1    LT-2
......................uggaucuuguucgauggcac......    122    0    JCK-1
......................uggCucuuguucgauggcac......    1      1    LT-1
......................uggaucuuguucgaNggcac......    1      1    LCK-1
......................uggaucuuguucgauggcac......    183    0    JCK-2
......................uggaucuuguucgauggcaA......    2      1    LT-1
......................uggaucuuguucgauggcaG......    1      1    JCK-1
......................uggaucuuguucgCuggcac......    3      1    LT-2
......................uggCucuuguucgauggcac......    1      1    LT-3
......................uggaucuuguucgauggcCc......    1      1    LT-3
......................uggaucuuguucgaCggcac......    1      1    LT-1
......................uggaucuuCuucgauggcac......    1      1    LT-2
......................uggaucuuguucgauggcCc......    1      1    LT-2
......................uggaucuuguucgaCggcac......    1      1    JCK-2
......................uggaucuuguucgNuggcac......    2      1    LCK-1
......................uggCucuuguucgauggcac......    1      1    JCK-3
......................uggaucuuguucgCuggcac......    1      1    JCK-1
......................uggCucuuguucgauggcac......    3      1    LCK-3
......................uggaucuuguucgauggcaA......    7      1    LT-2
......................uggaucuuguucgauggcaU......    4      1    JCK-3
......................uggaucuuguucgauggcaU......    1      1    JT-2
......................uggaucuuguucgauggcac......    916    0    LT-2
......................uggaucuuguucgauggcacC.....    11     1    LCK-3
......................uggCucuuguucgauggcacu.....    53     1    LT-2
......................uAgaucuuguucgauggcacu.....    2      1    JCK-3
......................uggaucuuguucgCuggcacu.....    18     1    JCK-3
......................ugCaucuuguucgauggcacu.....    1      1    LT-2
......................uggaucuuguucgauggcUcu.....    1      1    JT-2
......................uggaucuuguucUauggcacu.....    1      1    JCK-2
......................uggaucuuguucgauggcCcu.....    1      1    JT-1
......................uAgaucuuguucgauggcacu.....    4      1    LT-3
......................uggaucuuguucgauggcacG.....    6      1    JCK-3
......................uggaucuuguucgauggcacN.....    1      1    JT-2
......................uggaucuuguucgauggcCcu.....    6      1    JCK-3
......................uggaucuuguucAauggcacu.....    3      1    JCK-1
......................uggaucuuguCcgauggcacu.....    3      1    JCK-2
......................uggaucuuguucgauggcacN.....    1      1    JCK-1
......................uggaucuuguucgauggcacu.....    12438  0    JCK-3
......................uggaCcuuguucgauggcacu.....    2      1    JCK-2
......................uggaucuuguucgaGggcacu.....    2      1    LCK-1
......................uggaucuuguucgCuggcacu.....    18     1    LT-3
......................uggCucuuguucgauggcacu.....    6      1    LCK-1
```

```
                      Mature                                                        Star
uccaucuuguucguucacccucuggaucuuguucgauggcacuccccuuguauccaaucucucucuuucucucucucucucuuauggugcgaguggaagugccaugaacuugauccagaaggugcucucgaacaaguuacaccuacuuaauaucuacuuucuucuuucuccauuacuauuauuaaugaugguuucuuuuuucugauucugauuuuuaauuuauuuauuuguuuuuguguuuguguuu
......................Gggaucuuguucgauggcacu.............................................................................................................................................................................................................   1      1   LT-2
......................uggaucuuguucgauAgcacu.............................................................................................................................................................................................................   12     1   JCK-1
......................uCgaucuuguucgauggcacu.............................................................................................................................................................................................................   1      1   JCK-2
......................uggaucuuguuAgauggcacu.............................................................................................................................................................................................................   1      1   JCK-3
......................uggaucuuguucgauggcacu.............................................................................................................................................................................................................   712    0   JT-1
......................uggaucuuguucgauggcacu.............................................................................................................................................................................................................   1957   0   LCK-2
......................uggaucuuguucgauggcacA.............................................................................................................................................................................................................   34     1   LCK-1
......................uggaucuuguucgauggcacA.............................................................................................................................................................................................................   31     1   JCK-2
......................uggaucCuguucgauggcacu.............................................................................................................................................................................................................   1      1   LT-3
......................uggaucuuguucgCuggcacu.............................................................................................................................................................................................................   12     1   JCK-1
......................uggaucuuguucgauAgcacu.............................................................................................................................................................................................................   2      1   LT-3
......................uggaucAuguucgauggcacu.............................................................................................................................................................................................................   2      1   JCK-2
......................uNgaucuuguucgauggcacu.............................................................................................................................................................................................................   1      1   LCK-3
......................uggaucuuguucUauggcacu.............................................................................................................................................................................................................   4      1   JCK-1
......................uggaucuuguucAauggcacu.............................................................................................................................................................................................................   3      1   LT-2
......................uggaucuCguucgauggcacu.............................................................................................................................................................................................................   1      1   LT-1
......................uggaucuuguucgNuggcacu.............................................................................................................................................................................................................   12     1   LCK-1
......................uggaucuuguucgauggcacu.............................................................................................................................................................................................................   1998   0   LCK-1
......................uggaucuuguucgauggcacA.............................................................................................................................................................................................................   28     1   LT-2
......................uggaucuuguucgCuggcacu.............................................................................................................................................................................................................   2      1   JT-3
......................uggaucuuguucgauggcUcu.............................................................................................................................................................................................................   1      1   LT-1
......................uggaucuCguucgauggcacu.............................................................................................................................................................................................................   1      1   JCK-1
......................uggaucuuguucgauggcGcu.............................................................................................................................................................................................................   1      1   LT-2
......................uggaucuuguucgaugAcacu.............................................................................................................................................................................................................   4      1   JCK-1
......................uggaucuuguucgauggcaUu.............................................................................................................................................................................................................   14     1   LT-3
......................uggaucuuguucgauggcCcu.............................................................................................................................................................................................................   21     1   JCK-2
......................uggaucCuguucgauggcacu.............................................................................................................................................................................................................   4      1   JCK-3
......................uggCucuuguucgauggcacu.............................................................................................................................................................................................................   5      1   LCK-2
......................uggaucuuguucgauggcCcu.............................................................................................................................................................................................................   5      1   LCK-3
......................uggNucuuguucgauggcacu.............................................................................................................................................................................................................   1      1   LCK-1
......................uAgaucuuguucgauggcacu.............................................................................................................................................................................................................   1      1   JCK-1
......................uggaucuuAuucgauggcacu.............................................................................................................................................................................................................   3      1   LT-2
......................uggaucuuguucgauggcaAu.............................................................................................................................................................................................................   3      1   JCK-1
......................uNgaucuuguucgauggcacu.............................................................................................................................................................................................................   6      1   JCK-3
......................uggGucuuguucgauggcacu.............................................................................................................................................................................................................   5      1   JCK-1
......................uggCucuuguucgauggcacu.............................................................................................................................................................................................................   25     1   LT-3
......................uggCucuuguucgauggcacu.............................................................................................................................................................................................................   12     1   LCK-3
......................uggCucuuguucgauggcacu.............................................................................................................................................................................................................   37     1   JCK-2
......................uggaucuuguucgauggcacG.............................................................................................................................................................................................................   1      1   LCK-3
......................uggaucuuguucgauggcacA.............................................................................................................................................................................................................   61     1   LT-3
......................uggaucuuguucgauggcacG.............................................................................................................................................................................................................   9      1   LT-3
......................uggaucuuguucgauggUacu.............................................................................................................................................................................................................   2      1   JCK-1
......................uggaucuuguucgaugAcacu.............................................................................................................................................................................................................   3      1   LCK-3
......................uAgaucuuguucgauggcacu.............................................................................................................................................................................................................   1      1   LT-1
......................uggaucuuguucgauggcCcu.............................................................................................................................................................................................................   3      1   LT-3
......................uggaucuuguucgUuggcacu.............................................................................................................................................................................................................   1      1   LT-2
......................uggaucuuguucgauggcGcu.............................................................................................................................................................................................................   1      1   LCK-3
......................uggCucuuguucgauggcacu.............................................................................................................................................................................................................   1      1   JT-1
......................uggaucuuguucAauggcacu.............................................................................................................................................................................................................   1      1   LT-1
......................uggaucuuguucgaugAcacu.............................................................................................................................................................................................................   9      1   JCK-2
......................uggNucuuguucgauggcacu.............................................................................................................................................................................................................   10     1   JCK-1
......................Cggaucuuguucgauggcacu.............................................................................................................................................................................................................   4      1   JCK-2
......................uggaucuuguucgaugAcacu.............................................................................................................................................................................................................   8      1   LT-1
......................uggaucuuguucgCuggcacu.............................................................................................................................................................................................................   33     1   JCK-2
......................uggaucuuguucgauggcacu.............................................................................................................................................................................................................   5446   0   LT-2
......................uggaucuuguucgauggcacA.............................................................................................................................................................................................................   24     1   LCK-2
......................uggaucuuguCcgauggcacu.............................................................................................................................................................................................................   1      1   LT-2
......................uggaucuuguucgauggcacu.............................................................................................................................................................................................................   9876   0   JCK-1
......................uggCucuuguucgauggcacu.............................................................................................................................................................................................................   15     1   LT-1
......................uggaucuuguCcgauggcacu.............................................................................................................................................................................................................   1      1   LT-3
......................uggaucuuguucgauggcacu.............................................................................................................................................................................................................   6606   0   LCK-3
......................uggaucuuguuNgauggcacu.............................................................................................................................................................................................................   21     1   JCK-1
......................uggaucuuguucgauggcacu.............................................................................................................................................................................................................   288    0   JT-3
......................uggaucuuguucgauggcacu.............................................................................................................................................................................................................   181    0   JT-2
......................uggaCcuuguucgauggcacu.............................................................................................................................................................................................................   1      1   LCK-1
......................uggaCcuuguucgauggcacu.............................................................................................................................................................................................................   2      1   LT-1
......................uggaCcuuguucgauggcacu.............................................................................................................................................................................................................   2      1   JCK-3
......................uggaucuuAuucgauggcacu.............................................................................................................................................................................................................   2      1   LCK-3
......................uggaucuuguucgauggUacu.............................................................................................................................................................................................................   1      1   LT-3
......................uggaucuuguucgauggcaUu.............................................................................................................................................................................................................   23     1   LCK-1
```

```
                      Mature                                                                          Star
uccaucuuguucguucacccucuggaucuuguucgauggcacuccccuuguauccaaucucucucuuucucucucucucucucuuauggugcgaguggaagugccaugaacuugauccagaaggugcucucgaacaaguuacaccuacuuaauaucuacuuucuucuuucuccauuacuauuauuaaugaugguuucuuuuuucugauucugauuuuuaauuuauuuauuuguuuuuuguguuuguguuu
......................uNgaucuuguucgauggcacu...............................................................................................................................................................................   18     1    JCK-2
......................uggaucuuguucgauggNacu...............................................................................................................................................................................   1      1    JCK-3
......................uggaucuuguucgauggcaGu...............................................................................................................................................................................   1      1    JCK-1
......................uggaucuCguucgauggcacu...............................................................................................................................................................................   2      1    JCK-2
......................uggaucuuguucgCuggcacu...............................................................................................................................................................................   20     1    LT-2
......................uggaucuugCucgauggcacu...............................................................................................................................................................................   2      1    JCK-2
......................uggaucuuguucgaCggcacu...............................................................................................................................................................................   1      1    LT-2
......................uggauUuuguucgauggcacu...............................................................................................................................................................................   1      1    LT-2
......................uggaucuuguucgauggcaAu...............................................................................................................................................................................   1      1    LCK-3
......................uggaucuuguucgauggcacA...............................................................................................................................................................................   30     1    JCK-1
......................uggaucuuguucgauggcacA...............................................................................................................................................................................   100    1    LCK-3
......................uggaucuuguucgauggcaUu...............................................................................................................................................................................   10     1    LCK-3
......................uggaucuuguucgaugAcacu...............................................................................................................................................................................   1      1    JT-3
......................uggaucuuguuUgauggcacu...............................................................................................................................................................................   2      1    LT-1
......................uggaucuuguucgauggcaNu...............................................................................................................................................................................   8      1    JCK-1
......................Cggaucuuguucgauggcacu...............................................................................................................................................................................   1      1    LT-3
......................uggaucuuguucgauggcacG...............................................................................................................................................................................   5      1    LT-1
......................uggaucuuguucgGuggcacu...............................................................................................................................................................................   1      1    JCK-1
......................uUgaucuuguucgauggcacu...............................................................................................................................................................................   2      1    JCK-3
......................uggaucuuguucAauggcacu...............................................................................................................................................................................   1      1    LCK-3
......................uggaucuuUuucgauggcacu...............................................................................................................................................................................   3      1    JCK-3
......................uggaucuuguucgauggcaAu...............................................................................................................................................................................   2      1    LT-1
......................uAgaucuuguucgauggcacu...............................................................................................................................................................................   2      1    JCK-2
......................uggaucuuAuucgauggcacu...............................................................................................................................................................................   1      1    LT-3
......................uggaucuuguucgCuggcacu...............................................................................................................................................................................   3      1    LCK-2
......................uggaucuuguuUgauggcacu...............................................................................................................................................................................   1      1    JCK-1
......................uggaucuuguucgauggcacu...............................................................................................................................................................................   7444   0    LT-3
......................uggaucuuguucgauAgcacu...............................................................................................................................................................................   26     1    JCK-2
......................uggaucuuguucgauggcaUu...............................................................................................................................................................................   8      1    JCK-3
......................Cggaucuuguucgauggcacu...............................................................................................................................................................................   1      1    JCK-3
......................uggaucuuguucgaugAcacu...............................................................................................................................................................................   4      1    JCK-3
......................uggaucuuguucgauggcaAu...............................................................................................................................................................................   8      1    LT-2
......................uggaucuuguucCauggcacu...............................................................................................................................................................................   1      1    JCK-3
......................ugAaucuuguucgauggcacu...............................................................................................................................................................................   3      1    JCK-3
......................uggaucuuguuUgauggcacu...............................................................................................................................................................................   1      1    LCK-2
......................uggaucCuguucgauggcacu...............................................................................................................................................................................   3      1    JCK-1
......................uggauNuuguucgauggcacu...............................................................................................................................................................................   3      1    LCK-1
......................uggaucuuguucgauggcacG...............................................................................................................................................................................   2      1    LT-2
......................uggaucuuguCcgauggcacu...............................................................................................................................................................................   2      1    JCK-1
......................ugNaucuuguucgauggcacu...............................................................................................................................................................................   1      1    LCK-1
......................uggaucuuguucgaugUcacu...............................................................................................................................................................................   1      1    JCK-1
......................uggaucuuguucgauggcacA...............................................................................................................................................................................   12     1    LT-1
......................uggaucuuUuucgauggcacu...............................................................................................................................................................................   1      1    LT-1
......................uggaucuuguucgGuggcacu...............................................................................................................................................................................   1      1    JCK-3
......................uggaucuuguucgCuggcacu...............................................................................................................................................................................   1      1    JT-2
......................uggaucuugCucgauggcacu...............................................................................................................................................................................   1      1    JCK-1
......................uggaucuuguucgaugAcacu...............................................................................................................................................................................   5      1    LT-3
......................ugAaucuuguucgauggcacu...............................................................................................................................................................................   4      1    LT-2
......................uggCucuuguucgauggcacu...............................................................................................................................................................................   24     1    JCK-1
......................uggaucuuguucgauggUacu...............................................................................................................................................................................   6      1    JCK-2
......................uggaucuuAuucgauggcacu...............................................................................................................................................................................   1      1    JCK-3
......................uAgaucuuguucgauggcacu...............................................................................................................................................................................   1      1    LCK-2
......................uggaucuuguucgauggcNcu...............................................................................................................................................................................   6      1    JCK-1
......................ugAaucuuguucgauggcacu...............................................................................................................................................................................   3      1    LT-1
......................uggaucuuguucgCuggcacu...............................................................................................................................................................................   10     1    LT-1
......................uggaucuuguucgauggcacC...............................................................................................................................................................................   7      1    LT-2
......................uggaucuuguucgauggcaUu...............................................................................................................................................................................   10     1    LT-1
......................uggaucuuguucNauggcacu...............................................................................................................................................................................   1      1    LCK-1
......................uggaucuuguucgaCggcacu...............................................................................................................................................................................   2      1    JCK-1
......................uggaucCuguucgauggcacu...............................................................................................................................................................................   1      1    LCK-3
......................uggaucuuguucgauggcaAu...............................................................................................................................................................................   8      1    JCK-2
......................uggaucuuUuucgauggcacu...............................................................................................................................................................................   2      1    LT-3
......................uggaucuuguucgauggGacu...............................................................................................................................................................................   4      1    JCK-1
......................uggaucuuAuucgauggcacu...............................................................................................................................................................................   1      1    JCK-2
......................uggaucuuguucgauggAacu...............................................................................................................................................................................   1      1    LCK-3
......................uggaucuuguucgauggcCcu...............................................................................................................................................................................   6      1    LT-1
......................Cggaucuuguucgauggcacu...............................................................................................................................................................................   1      1    LT-1
......................uUgaucuuguucgauggcacu...............................................................................................................................................................................   2      1    LT-2
......................uggaucuuguucgauggcacG...............................................................................................................................................................................   7      1    JCK-2
......................uggaucuuguucAauggcacu...............................................................................................................................................................................   12     1    JCK-2
```

Mature **Star**

uccaucuuguucguucacccucuggaucuuguucgauggcacuccccuuguauccaaucucucucuuucucucucucucucuuauggugcgaguggaagugccaugaacuugauccagaaggugcucucgaacaaguuacaccuacuuaauaucuacuuucuucuuucuccauuacuauuauuaaugaugguuucuuuuucugauucugauuuuuaauuuauuuauuuguuuuuguguuuguguuu

uggaucuuguucgauggcacu	6201	0	LT-1
uggaucuuguucgauggAacu	1	1	JCK-1
uggaucuuguucgaNggcacu	5	1	LCK-1
uggGucuuguucgauggcacu	2	1	LT-3
uggaucuuguucgauggUacu	5	1	JCK-3
uggaucuuguucAauggcacu	2	1	JCK-3
uggaucuuguucgCuggcacu	11	1	LCK-3
uUgaucuuguucgauggcacu	3	1	JCK-2
uggaucuuguuGgauggcacu	2	1	JCK-1
uAgaucuuguucgauggcacu	3	1	LCK-3
uggGucuuguucgauggcacu	1	1	JCK-2
uggaucCuguucgauggcacu	6	1	JCK-2
uggaucuuguucgauggcGcu	1	1	JCK-2
uggaucuuguucgauggcaAu	1	1	JCK-3
uggaucuuguucgauggcCcu	12	1	LT-2
uggaucuuguucgauggcaUu	10	1	JCK-1
uggauUuuguucgauggcacu	3	1	JCK-2
uggaucuuNuucgauggcacu	1	1	JCK-3
uggCucuuguucgauggcacu	22	1	JCK-3
Gggaucuuguucgauggcacu	1	1	LT-3
uggaucuuguucgauggcaUu	8	1	LT-2
uggaucuuguuUgauggcacu	8	1	JCK-2
uggaucuuguucgauggcCcu	10	1	JCK-1
uggaCcuuguucgauggcacu	1	1	LCK-2
Cggaucuuguucgauggcacu	1	1	JT-1
ugAaucuuguucgauggcacu	3	1	LCK-3
ugAaucuuguucgauggcacu	8	1	JCK-2
uggaucuuguuAgauggcacu	2	1	JCK-1
uggaucuuguGcgauggcacu	2	1	JCK-1
uggaucuuguucgauggcacA	25	1	JCK-3
uggaucuuguuUgauggcacu	4	1	LT-3
uggaucuuguucgauggcacG	1	1	LCK-1
uggaucuuguucgCuggcacu	1	1	LCK-1
Cggaucuuguucgauggcacu	1	1	LT-2
uggaucuuguucgauggcacu	17137	0	JCK-2
uggaucuuguucgauggcacC	15	1	LT-1
uggaucuuguucAauggcacu	3	1	LT-3
uggaucuuguucgaCggcacu	1	1	LCK-3
uggaucuuguucgauggcacC	15	1	LT-3
uggaucuuguucgaugUcacu	1	1	LT-3
uNgaucuuguucgauggcacu	10	1	JCK-1
uggaucuuguucgaugUcacu	2	1	JCK-2
uggaucuuguucgCuggcacu	4	1	JT-1
uggGucuuguucgauggcacu	1	1	LT-2
uggaucuugCucgauggcacu	1	1	LT-1
uggaucuuguuUgauggcacu	9	1	JCK-3
uggaucuuguucgauggcCcu	2	1	LCK-1
uggaucuuguucgaCggcacu	1	1	LT-1
uggaucuuguucgauggcUcu	1	1	LT-3
ugAaucuuguucgauggcacu	2	1	LT-3
uggaucuuguucgauAgcacu	11	1	JCK-3
uggaucuuguucgauAgcacu	2	1	LT-1
uggaucuuguucgauggcacG	7	1	JCK-1
uggaucuuguucgauggcacC	3	1	JCK-1
uNgaucuuguucgauggcacu	1	1	LCK-1
uggaucuuguucgauggcaUu	21	1	LCK-2
uggaucuuguucgauggcacC	7	1	JCK-2
ugNaucuuguucgauggcacu	8	1	LT-2
uggaucuuguucgauggcGcu	1	1	JCK-3
uggaucuuguucgauggcaUu	12	1	JCK-2
Cggaucuuguucgauggcacu	4	1	LCK-3
uggaucuuguucUauggcacu	4	1	JCK-3
Gggaucuuguucgauggcacu	2	1	JCK-2
uggaucuuguucgaCggcacu	1	1	LCK-2
uggaucuuguucgauggcacC	12	1	JCK-3
uggaucuuguucgaCggcacu	10	1	JCK-3
uAgaucuuguucgauggcacu	4	1	LT-2
uggaCcuuguucgauggcacu	1	1	LT-2
uggaucuuguucgaCggcacu	5	1	JCK-2
ugAaucuuguucgauggcacu	1	1	JCK-1

Mature **Star**

```
uccaucuuguucguucacccucuggaucuuguucgauggcacuccccuuguauccaaucucucucuuucucucucucucucuuauggugcgaguggaagugccaugaacuugauccagaaggugcucucgaacaaguuacaccuacuuaauaucuacuuucuucuuucuccauuacuauuauuaaugaugguuucuuuuuucugauucugauuuuuaauuuauuuauuuguuuuuguguuuguguuu
```

......................uggaucuuguucgauggcCcu	2	1	LCK-2
......................uggaucuuguucgauggcaAuc	1	1	JCK-3
......................uggaucuuguucgauggcacAc	1	1	JCK-1
......................uggaucuuguucgauggcacNc	1	1	JCK-1
......................uggaucuuguucgauggUacuc	1	1	LT-1
......................uggaucuuguucgauggcacuG	4	1	LT-2
......................uggaucuuguucgauggcCcuc	7	1	LT-2
......................uggaucuuguucgCuggcacuc	17	1	LT-1
......................uNgaucuuguucgauggcacuc	15	1	JCK-1
......................uggaucuuguucgauggcacuA	53	1	JCK-3
......................uggaucuuguucgauAgcacuc	10	1	JCK-1
......................Nggaucuuguucgauggcacuc	1	1	JCK-3
......................uggaucuuguuUgauggcacuc	7	1	JCK-3
......................Cggaucuuguucgauggcacuc	1	1	JCK-1
......................uggaucuuguucgauggAacuc	1	1	LT-1
......................uggaucuuguucgauggcCcuc	7	1	JCK-1
......................uAgaucuuguucgauggcacuc	1	1	LT-2
......................uggaucuuguucgauggcacAc	1	1	JCK-2
......................uggaucuuguucgauggcacuc	3507	0	LT-2
......................uggaucuuguucgauggcacuG	8	1	JCK-1
......................uggaucuuguucgauggNacuc	2	1	JCK-3
......................uggaucuuguucgauggUacuc	1	1	LT-3
......................uggaucGuguucgauggcacuc	1	1	LT-1
......................uggaucuuguucgaugCcacuc	1	1	JCK-1
......................uggaucuuguucgauggcacuA	18	1	LCK-2
......................uAgaucuuguucgauggcacuc	3	1	JCK-2
......................uggaucuuguucUauggcacuc	3	1	LT-1
......................uggaucuuguucgauggcacGc	2	1	JCK-2
......................uggaucuuguuNgauggcacuc	1	1	JT-1
......................uggaucuuguucgauggcaUuc	3	1	LT-1
......................uggaucuuguucgauggcacCc	2	1	JCK-2
......................uggaucuuguucgaugUcacuc	1	1	JCK-2
......................uggaucuuguucgauggcacuc	356	0	JT-1
......................uggaucuuNuucgauggcacuc	2	1	JCK-3
......................uggaucuuguucgauggcacuc	825	0	LCK-2
......................uggaucuuguucgauggcacCc	3	1	JCK-1
......................uggaucuuguucgauggcacCc	2	1	LT-1
......................uggaucuuguucgCuggcacuc	10	1	LT-2
......................uggaucuuguucgauggcacuU	98	1	JCK-3
......................uggaucuuguucgauggcNcuc	5	1	JCK-1
......................uggaucuuguucgauggcacuc	11364	0	JCK-1
......................uggaucuuguucUauggcacuc	2	1	LT-3
......................ugAaucuuguucgauggcacuc	1	1	LCK-1
......................uggaucuuguucAauggcacuc	1	1	LT-1
......................uggaucuuguucgaugAcacuc	2	1	JCK-2
......................uggaucuuguucgauggcacuc	785	0	LCK-1
......................uggaucuuguucgCuggcacuc	33	1	JCK-2
......................Cggaucuuguucgauggcacuc	5	1	JCK-3
......................uggaucuuguucgauggUacuc	6	1	JCK-3
......................uggaucuuguucgauggcacuc	3568	0	LCK-3
......................uggCucuuguucgauggcacuc	30	1	JCK-2
......................uggaucuuUuucgauggcacuc	1	1	JCK-3
......................uggaucuuguucgauggcacuN	1	1	LT-2
......................uggaucuuguucgauggcacuU	147	1	JCK-2
......................uggCucuuguucgauggcacuc	29	1	JCK-1
......................uggaucuuguucgauggcacuU	147	1	LT-1
......................uggaucuuguucgauggcaUuc	4	1	LCK-3
......................uggaucuuguucgauggcacuA	17	1	LT-2
......................Cggaucuuguucgauggcacuc	1	1	LT-3
......................uggaucuuguucgCuggcacuc	17	1	JCK-3
......................uggGucuuguucgauggcacuc	2	1	JCK-3
......................uggaucuuguAcgauggcacuc	1	1	JCK-1
......................uggaucuuguucgCuggcacuc	4	1	LCK-3
......................uggaucuuguuAgauggcacuc	1	1	LT-3
......................uggaucuuguucgauCgcacuc	1	1	JCK-2
......................uggaucuuguucgauggcacAc	1	1	LT-3
......................uggaucuCguucgauggcacuc	1	1	JCK-3
......................uggaucuuAuucgauggcacuc	4	1	JCK-2
......................uggaucuuguucAauggcacuc	4	1	JCK-3
......................uggauUuuguucgauggcacuc	10	1	JCK-3

Mature Star

uccaucuuguucguucacccucuggaucuuguucgauggcacuccccuuguauccaaucucucucuuucucucucucucucuuauggugcgaguggaagugccaugaacuugauccagaaggugcucucgaacaaguuacaccuacuuaauaucuacuuucuucuuucuccauuacuauuauuaaugaugguuucuuuuucugauucugauuuuaauuuauuuauuuguuuuuguguuuguguuu

......................uggaucuuguucgauggcacCc	4	1	JCK-3
......................uggaucuuguuUgauggcacuc	3	1	JCK-1
......................uNgaucuuguucgauggcacuc	7	1	JCK-3
......................uggaucuuAuucgauggcacuc	1	1	LCK-1
......................uggaCcuuguucgauggcacuc	1	1	LCK-1
......................uggaucuuguucAauggcacuc	3	1	LT-3
......................uggaucuuguucgaCggcacuc	2	1	JCK-2
......................uggaucuuguucgaugAcacuc	2	1	LT-3
......................uggaucuuUuucgauggcacuc	2	1	JCK-1
......................ugAaucuuguucgauggcacuc	1	1	LCK-3
......................uggaucuuguucgaugCcacuc	2	1	JCK-3
......................uggaCcuuguucgauggcacuc	6	1	JCK-2
......................uUgaucuuguucgauggcacuc	3	1	LT-3
......................uggaucuuguucgaNggcacuc	1	1	LCK-1
......................uggaucuuguuUgauggcacuc	10	1	JCK-2
......................uggaucuuguucUauggcacuc	5	1	JCK-2
......................Cggaucuuguucgauggcacuc	1	1	LT-1
......................Cggaucuuguucgauggcacuc	2	1	JCK-2
......................uggaucuuguucgauggcaUuc	33	1	JCK-2
......................uggaucuuAuucgauggcacuc	1	1	JCK-1
......................uggaucuuguucgauggcCcuc	11	1	LT-3
......................uggaucNuguucgauggcacuc	1	1	JCK-3
......................uggaucuugCucgauggcacuc	2	1	LT-2
......................uggaucuuguucgauggcaUuc	19	1	JCK-1
......................uggaucuuguucgauggcacAc	1	1	JCK-3
......................uAgaucuuguucgauggcacuc	1	1	LCK-3
......................uggaucuuguucgauggcacuN	6	1	JCK-1
......................uggaucuuguucgaCggcacuc	1	1	LT-1
......................uggaCcuuguucgauggcacuc	3	1	JCK-1
......................uggaucCuguucgauggcacuc	5	1	JCK-2
......................uggaucuuAuucgauggcacuc	1	1	JCK-3
......................uggaucuuguucAauggcacuc	3	1	LT-2
......................uggaucuuguucgaCggcacuc	2	1	LT-2
......................uggaucuuguGcgauggcacuc	5	1	JCK-1
......................uggaucuuguucgauggcacCc	1	1	JT-1
......................uggaucuuguucgauggcacuA	17	1	LCK-1
......................uggaucuuguucAauggcacuc	3	1	JCK-1
......................uggaucuuguucgaugUcacuc	2	1	JCK-3
......................uggGucuuguucgauggcacuc	1	1	JCK-2
......................uggaucuCguucgauggcacuc	1	1	JCK-1
......................uggaucuuUuucgauggcacuc	1	1	JCK-2
......................uggaucuuguucgauggcacuU	30	1	LCK-2
......................uggaucuugGucgauggcacuc	1	1	LT-1
......................uggaucuuguucgauggcaNuc	9	1	JCK-1
......................uggCucuuguucgauggcacuc	22	1	JCK-3
......................ugNaucuuguucgauggcacuc	10	1	LT-2
......................uggaucuuAuucgauggcacuc	2	1	LT-1
......................uggaucuuguuNgauggcacuc	18	1	JCK-1
......................uggaucuuguucgauggcaAuc	1	1	LT-3
......................uggaucuuguucgCuggcacuc	1	1	JT-1
......................ugAaucuuguucgauggcacuc	2	1	LT-2
......................uggaCcuuguucgauggcacuc	1	1	JCK-3
......................uggaucuuUuucgauggcacuc	1	1	LT-3
......................uggaucuugCucgauggcacuc	2	1	LT-1
......................uggaucuuguucgauggcCcuc	24	1	JCK-2
......................uggaucuuguucgauggcacuc	177	0	JT-3
......................uggaucuuguucgauggcacuA	72	1	JCK-2
......................ugAaucuuguucgauggcacuc	3	1	JCK-1
......................ugAaucuuguucgauggcacuc	2	1	LT-1
......................uggaucuuguucgauggcacuc	7340	0	LT-1
......................uggaucuuguucgauggcacuc	143	0	JT-2
......................uggaucuugCucgauggcacuc	2	1	JCK-2
......................uAgaucuuguucgauggcacuc	4	1	LT-3
......................uggauAuuguucgauggcacuc	1	1	JCK-3
......................uggCucuuguucgauggcacuc	8	1	LCK-3
......................uggaucuuguucgaugAcacuc	2	1	JCK-3
......................uggaucuuguucgauggcCcuc	2	1	LCK-1
......................uggaucuuguucgauggcGcuc	12	1	JT-1
......................uggaucuuguucgauggcaUuc	6	1	LT-2
......................uggaucCuguucgauggcacuc	5	1	LCK-3

```
                      Mature                                                                            Star
uccaucuuguucguucacccucuggaucuuguucgauggcacuccccuuguauccaaucucucucuuucucucucucucucuuauggugcgaguggaagugccaugaacuugauccagaaggugcucucgaacaaguuacaccuacuuaauaucuacuuucuucuuucuccauuacuauuauuaaugaugguuucuuuuuucugauucugauuuuuaauuuauuuauuuguuuuuguguuuguguuu
......................uggaucuuguucgauggcacuA.............................................................................................................................................................................................................  37     1  JCK-1
......................uggaucuuguucgauggcacuU.............................................................................................................................................................................................................  19     1  LCK-1
......................uggCucuuguucgauggcacuc.............................................................................................................................................................................................................  1      1  JT-1
......................uggaucuuguucgauggcCcuc.............................................................................................................................................................................................................  11     1  JCK-3
......................uggaucuuguuGgauggcacuc.............................................................................................................................................................................................................  2      1  JCK-1
......................uggaucuuguucgauAgcacuc.............................................................................................................................................................................................................  42     1  JCK-2
......................uggCucuuguucgauggcacuc.............................................................................................................................................................................................................  3      1  LCK-2
......................uggaucuuguucgauggcCcuc.............................................................................................................................................................................................................  6      1  LT-1
......................uggaucuuguucgauggcacuA.............................................................................................................................................................................................................  44     1  LT-3
......................uggauUuuguucgauggcacuc.............................................................................................................................................................................................................  10     1  JCK-2
......................uggaucuuguucgaCggcacuc.............................................................................................................................................................................................................  1      1  JCK-1
......................uggaucuuguucAauggcacuc.............................................................................................................................................................................................................  8      1  JCK-2
......................uggaucuuguucgauUgcacuc.............................................................................................................................................................................................................  3      1  JCK-3
......................uggCucuuguucgauggcacuc.............................................................................................................................................................................................................  29     1  LT-3
......................uggCucuuguucgauggcacuc.............................................................................................................................................................................................................  34     1  LT-2
......................uggaucuuguucgCuggcacuc.............................................................................................................................................................................................................  27     1  LT-3
......................uggaucuuguucgauggUacuc.............................................................................................................................................................................................................  2      1  JCK-1
......................uggaucuuguCcgauggcacuc.............................................................................................................................................................................................................  1      1  JCK-3
......................uggaucCuguucgauggcacuc.............................................................................................................................................................................................................  1      1  LT-1
......................uggaucuuguucgauggcacuU.............................................................................................................................................................................................................  72     1  LT-2
......................uggaucuuguucgCuggcacuc.............................................................................................................................................................................................................  1      1  LCK-2
......................uggaucuuguucgGuggcacuc.............................................................................................................................................................................................................  1      1  LT-2
......................uggaucuuguucgNuggcacuc.............................................................................................................................................................................................................  2      1  LCK-1
......................uggaucuuguucgauggcaUuc.............................................................................................................................................................................................................  6      1  LT-3
......................uggaucuuguucgauggcacCc.............................................................................................................................................................................................................  2      1  LT-3
......................uggaucuuguucgauAgcacuc.............................................................................................................................................................................................................  1      1  LT-3
......................uUgaucuuguucgauggcacuc.............................................................................................................................................................................................................  3      1  LT-2
......................uggaucuuguucgauggcGcuc.............................................................................................................................................................................................................  3      1  LCK-3
......................uggaucuuguCcgauggcacuc.............................................................................................................................................................................................................  2      1  JCK-1
......................Gggaucuuguucgauggcacuc.............................................................................................................................................................................................................  1      1  JCK-2
......................uggCucuuguucgauggcacuc.............................................................................................................................................................................................................  16     1  LT-1
......................uggNucuuguucgauggcacuc.............................................................................................................................................................................................................  6      1  JCK-1
......................uggauAuuguucgauggcacuc.............................................................................................................................................................................................................  1      1  JCK-2
......................uggaucuuguucgauggUacuc.............................................................................................................................................................................................................  2      1  LT-2
......................uggaucuuguucgauAgcacuc.............................................................................................................................................................................................................  26     1  JCK-3
......................uggaucuuguucgauggcacuU.............................................................................................................................................................................................................  113    1  LCK-3
......................uggaucuuguucgauggUacuc.............................................................................................................................................................................................................  4      1  JCK-2
......................uggaucuuguuUgauggcacuc.............................................................................................................................................................................................................  1      1  LT-2
......................uggaucuuguucgauggcaUuc.............................................................................................................................................................................................................  21     1  JCK-3
......................uAgaucuuguucgauggcacuc.............................................................................................................................................................................................................  3      1  JCK-3
......................uggaucuuguucgauggcacuU.............................................................................................................................................................................................................  82     1  JCK-1
......................uggaCcuuguucgauggcacuc.............................................................................................................................................................................................................  1      1  LT-3
......................uggaucuuguucgauggcacuc.............................................................................................................................................................................................................  14328  0  JCK-3
......................uUgaucuuguucgauggcacuc.............................................................................................................................................................................................................  3      1  JCK-2
......................uggaucuuguucgaCggcacuc.............................................................................................................................................................................................................  4      1  JCK-3
......................uggCucuuguucgauggcacuc.............................................................................................................................................................................................................  1      1  LCK-1
......................uggaucuuguucgauggcacuA.............................................................................................................................................................................................................  38     1  LT-1
......................uggGucuuguucgauggcacuc.............................................................................................................................................................................................................  1      1  LT-2
......................uggaucuuguucgauggcacuA.............................................................................................................................................................................................................  58     1  LCK-3
......................uNgaucuuguucgauggcacuc.............................................................................................................................................................................................................  25     1  JCK-2
......................uggaucuuguucgCuggcacuc.............................................................................................................................................................................................................  2      1  LCK-1
......................ugAaucuuguucgauggcacuc.............................................................................................................................................................................................................  3      1  JCK-2
......................uggaucuuguucgauAgcacuc.............................................................................................................................................................................................................  2      1  LT-1
......................uggaucuuguuUgauggcacuc.............................................................................................................................................................................................................  2      1  LT-1
......................uggaucuuguucgauggcaGuc.............................................................................................................................................................................................................  1      1  JCK-1
......................uggauUuuguucgauggcacuc.............................................................................................................................................................................................................  5      1  JCK-1
......................uggaucuuguucgauggGacuc.............................................................................................................................................................................................................  4      1  JCK-1
......................uggaucuuguucgauggcacuc.............................................................................................................................................................................................................  19469  0  JCK-2
......................uAgaucuuguucgauggcacuc.............................................................................................................................................................................................................  5      1  LT-1
......................ugCaucuuguucgauggcacuc.............................................................................................................................................................................................................  1      1  LT-2
......................ugAaucuuguucgauggcacuc.............................................................................................................................................................................................................  4      1  JCK-3
......................ugAaucuuguucgauggcacuc.............................................................................................................................................................................................................  1      1  LT-3
......................uggGucuuguucgauggcacuc.............................................................................................................................................................................................................  3      1  JCK-1
......................uggaucuuguucgauggcacuU.............................................................................................................................................................................................................  121    1  LT-3
......................uggaucuuguucgauggcCcuc.............................................................................................................................................................................................................  3      1  LCK-3
......................uggaucuuguucgaugAcacuc.............................................................................................................................................................................................................  6      1  JCK-1
......................uggaucuuguucUauggcacuc.............................................................................................................................................................................................................  1      1  JCK-3
......................uggaucuuguucgaugNcacuc.............................................................................................................................................................................................................  2      1  LT-2
......................uggaucuuguucgauggcacGc.............................................................................................................................................................................................................  1      1  JCK-3
......................uggaucCuguucgauggcacuc.............................................................................................................................................................................................................  1      1  JCK-1
```

Mature / Star			
uccaucuuguucguucacccucuggaucuuguucgauggcacuccccuuguauccaaucucucucuuucucucucucucucuuauggugcgaguggaagugccaugaacuugauccagaaggugcucucgaacaaguuacaccuacuuaauaucuacuuucuucuuucuccauuacuauuauuaaugaugguuucuuuuuucugauucugauuuuuaauuuauuuauuuguuuuuguguuuguguuu			
......................uggaucuuguucgCuggcacuc	16	1	JCK-1
......................uAgaucuuguucgauggcacuc	4	1	JCK-1
......................uggauAuuguucgauggcacuc	1	1	LT-1
......................Cggaucuuguucgauggcacuc	1	1	LT-2
......................uggaucuuguucgauggcacuc	8004	0	LT-3
......................uggaucuugCucgauggcacuc	1	1	JCK-3
......................uggaucuuguucgauggcacucA	23	1	LCK-3
......................uggaucuuguucgauggcacucc	12	0	LT-2
......................uggaucuuguucgauggcacucU	125	1	JCK-2
......................uggaucuuguucgauggcacucG	1	1	LCK-3
......................uggaucuuguucgauggcacucG	1	1	LT-3
......................uggaucuuguucgauggcacuUc	2	1	LT-3
......................uggaucuuguucgauggcacucc	28	0	LT-1
......................uggaucuuguucgauggcacucc	18	0	JCK-3
......................uggaucuuguucgauggcacucc	16	0	JCK-1
......................uggaucuuguucgauggcacucA	6	1	JT-3
......................uggaucuuguucgauggcacucA	33	1	LT-3
......................uggaucuuguucgauggcacucA	55	1	JCK-3
......................uggaucuuguucgauggcacucA	41	1	JCK-1
......................uggaucuuguucgauggcacucA	28	1	LT-2
......................uggaucuuguucgauggcacucU	88	1	LCK-3
......................uggaucuuguucgauggcacucA	56	1	LT-1
......................uggaucuuguucgauggcacucU	89	1	JCK-3
......................uggaucuuguucgauggcacucU	59	1	LT-1
......................uggaucuuguucgauggcacuAc	1	1	LT-1
......................uggaucuuguucgauggcacucA	70	1	JCK-2
......................uggaucuuguucgauggcacuUc	1	1	JCK-1
......................uggaucuuguucgauggcacucU	72	1	JCK-1
......................uUgaucuuguucgauggcacucc	1	1	JCK-3
......................uggaucuuguucgauggcacucU	12	1	JT-2
......................uggaucuuguucgauggcacucU	10	1	JT-1
......................uggaucuuguucgauggcacucG	1	1	JCK-3
......................uggaucuuguucgauggcacucU	6	1	JT-3
......................uggaucuuguucgauggcacuUc	2	1	LT-2
......................uggaucuuguucgauggcacucG	1	1	LT-1
......................uggaucuuguucgauggcacucc	7	0	LCK-3
......................uggaucuuguucgauggcacucc	34	0	JCK-2
......................uggaucuuguucgauggcacucA	4	1	JT-2
......................uggaucuuguucgauggcacucG	3	1	JCK-2
......................uggaucuuguucgauggcacucU	67	1	LT-2
......................uggaucuuguucgauggcacuAc	1	1	LT-2
......................uggaucuuguucgauggcacucU	49	1	LT-3
......................uggaucuuguucgauggcacucc	21	0	LT-3
......................uggaucuuguucgauggcacuccU	5	1	LCK-3
......................uggaucuuguucgauggcacuccU	4	1	LT-1
......................uggaucuuguucgauggcacucAc	10	1	LT-2
......................uggaucuuguucgauggcacuccc	4	0	LCK-3
......................uggaucuuguucgauggcacucGc	3	1	LT-1
......................uggaucuuguucgauggcacucAc	4	1	LT-1
......................uggaucuuguucgauggcacucAc	7	1	LCK-3
......................uggaucuuguucgauggcacuccU	3	1	LT-3
......................uggaucuuguucgauggcacucUc	1	1	LT-2
......................uggaucuuguucgauggcacucGc	2	1	LT-2
......................uggaucuuguucgauggcacuccc	2	0	LT-1
......................uggaucuuguucgauggcacuccU	10	1	LT-2
......................uggaucuuguucgauggcacuccAc	1	1	JCK-3
......................uggaucuuguucgauggcacucccU	1	1	LT-2
......................uggaucuuguucgauggcacuccAc	4	1	LT-2
......................uggaucuuguucgauggcacucUcc	4	1	LCK-3
......................uggaucuuguucgauggcacuccAc	2	1	JCK-2
......................uggaucuuguucgauggcacucccc	1	0	LT-3
......................uggaucuuguucgauggcacuccAc	2	1	JCK-1
......................uggaucuuguucgauggcacuccAc	5	1	LCK-3
......................uggaucuuguucgauggcacucccU	1	1	LT-1
......................uggaucuuguucgauggcacuccAc	1	1	LT-1
......................uggaucuuguucgauggcacucUccu	1	1	LT-1
.......................ggaucuuguucgauggca	5	0	JT-1
.......................ggaucuuguucgauggcacu	7	0	LT-1
.......................ggaucuuguucgauggcacu	3	0	JCK-3
.......................ggaucuuguucgauggcacu	6	0	LT-2

```
                      Mature                                                       Star
uccaucuuguucguucacccucuggaucuuguucgauggcacuccccuuguauccaaucucucucuuucucucucucucucucuuauggugcgaguggaagugccaugaacuugauccagaaggugcucucgaacaaguuacaccuacuuaauaucuacuuucuucuuucuccauuacuauuauuaaugaugguuucuuuuuucugauucugauuuuuaauuuauuuauuuguuuuuguguuuguguuu
.......................ggaucuuguucgauggcacu          4    0    JCK-2
.......................ggaucuuguucgauggcacu          3    0    LCK-3
.......................ggaucuuguucgauggcacu          1    0    JCK-1
.......................Ugaucuuguucgauggcacu          1    1    JCK-1
.......................ggaucuuguucgauggcacu          10   0    LT-3
.......................ggaucuuguucgauggcacuc         2    0    LT-1
.......................ggaucuuguucgauggcacuc         2    0    JCK-2
.......................ggaucuuguucgauggcacuc         3    0    LT-3
.......................ggaucuuguucgauggcacuc         6    0    JCK-1
.......................ggaucuuguucgauggcacuc         2    0    LCK-3
.......................ggaucuuguucgauggcacuc         3    0    JCK-3
.......................ggaucuuguucgauggcacucc        1    0    LT-1
.......................ggaucuuguucgauggcacucc        1    0    JCK-2
.......................ggaucuuguucgauggcacucc        1    0    JCK-3
.......................ggaucuuguucgauggcacucc        3    0    JCK-1
.......................ggaucuuguucgauggcacuccc       3    0    JCK-3
.......................ggaucuuguucgauggcacuccc       4    0    JCK-1
........................gaucuuguucgauggcacu          2    0    LT-2
........................gaucuuguucgauggcacu          2    0    JCK-1
........................gaucuuguucgauggcacu          1    0    JCK-3
........................gaucuuguucgauggcacuc         1    0    JCK-2
........................gaucuuguucgauggcacuc         1    0    LT-3
........................gaucuuguucgauggcacuc         4    0    LT-2
........................gaucuuguucgauggcacucc        12   0    JCK-2
........................gaucuuguucgauggcacucc        1    0    LT-3
........................gaucuuguucgauggcacucc        8    0    JCK-3
........................gaucuuguucgauggcacucc        1    0    LT-2
........................gaucuuguucgauggcacucc        5    0    JCK-1
........................Aaucuuguucgauggcacuccc       1    1    JCK-2
........................gaucuuguucgauggcacuccc       56   0    JCK-2
........................gaucuuguucgauggcacuccc       2    0    LT-1
........................gaucuuguucgauggcacuccU       1    1    JCK-3
........................gaucuuguucgauggcacuccc       6    0    LT-3
........................gaucuuguucgauggcacuccc       5    0    LCK-3
........................gaucuuguucgauggcacuccc       6    0    LT-2
........................gaucuuguucgauggcacuccc       37   0    JCK-3
........................gaucuuguucgauggcacuccc       24   0    JCK-1
.........................aucuuguucgauggcacu          1    0    LT-2
.........................aucuuguucgauggcacuc         1    0    JCK-3
.........................aucuuguucgauggcacuc         3    0    LT-3
.........................aucuuguucgauggcacuc         1    0    JCK-1
.........................aucuuguucgauggcacucU        1    1    LT-1
.........................aucuuguucgauggcacucU        1    1    JCK-3
.........................aucuuguucgauggcacucc        1    0    JCK-3
.........................aucuuguucgauggcacucc        1    0    JCK-2
.........................aucuuguucgauggcacucc        3    0    LT-3
.........................aucuuguucgauggcacucc        2    0    LT-2
.........................aucuuguucgauggcacucc        1    0    JCK-1
.........................aucuuguucgauggcacucU        2    1    JCK-2
.........................aucCuguucgauggcacuccc       1    1    LT-3
.........................aucuuguucgauggcacuccc       1    0    LT-1
.........................aucuuguucgauggcacuccc       2    0    JCK-3
.........................aucuuguucgauggcacuccc       5    0    JCK-2
.........................aucuuguucgauggcacuccc       1    0    LT-3
.........................aucuuguucgauggcacucccc      2    0    LT-3
..........................ucuuguucgauggcacuc         1    0    JCK-3
..........................ucuuguucgauggcacucc        4    0    LCK-3
..........................ucuuguucgauggcacuccc       2    0    LT-3
........................................................aucuUucucuuucucucucu          1    1    LT-3
.........................................................ucucucucuuucucucuUucucu      1    1    LT-3
.........................................................ucucucucuuucucucucuUucuc     1    1    JCK-3
.........................................................ucucucucuuucucucucuUucuc     1    1    JCK-1
.........................................................ucucucucuuucucucucuUucuc     1    1    JCK-2
.........................................................ucucucucCuucucucucucucucu    2    1    JCK-3
.........................................................ucucucucCuucucucucucucucu    2    1    JCK-1
..........................................................cucucucuuucucucucucuGu     1    1    LT-1
..........................................................cucucucuuucucuAucucucucucu 2    1    LT-1
...........................................................ucucucuuucucucAcuc       2    1    LCK-3
............................................................cuAucuuucucucucucucuc   1    1    LT-1
.............................................................ucucuuucuGucucucuc     5    1    LT-2
```

Mature **Star**

uccaucuuguucguucacccucuggaucuuguucgauggcacuccccuuguauccaaucucucucuuucucucucucucucuuauggugcgaguggaagugccaugaacuugauccagaaggugcucucgaacaaguuacaccuacuuaauaucuacuuucuucuuucucccauuacuauuauuaaugaugguuucuuuuuucugauucugauuuuuaauuuauuuauuuguuuuuguguuuguguuu

cuuuGucucucucucucu	1	1	LT-3
cuuucuUucucucucucuu	1	1	JCK-3
ucuAucucucucucucuu	2	1	LT-2
uggugcgaguggaagugccaugaacu	1	0	LT-3
gugcgaguggaagugccaugaacuug	5	0	LCK-3
gcgaguggaagugccaugaacuugau	2	0	LCK-3
cgaguggaagugccaugaacuugauccaga	1	0	LT-1
cgaguggaagugccaugaacuugauccagC	1	1	LT-1
gaguggaagugccaugaacuugaucc	1	0	JCK-3
gaguggaagugccaugaacuugaucc	1	0	JCK-2
gaguggaagugccaugaacuugauccaga	1	0	JCK-2
gaguggaagugccaugaacuugauccaga	1	0	JCK-1
gaguggaagugccaugaacuugauccaga	2	0	JCK-3
gaguggaagugccaugaacuugauccagaa	1	0	JCK-1
gaguggaagugccaugaacuugauccagaa	1	0	JCK-3
aguggaagugccaugaacuuga	1	0	LT-3
aguggaagugccaugaacuugauc	1	0	JCK-2
aguggaagugccaugaacuugauc	1	0	JCK-1
aguggaagugccaugaacuugauccaga	4	0	JCK-2
aguggaagugccaugaacuugauccaga	2	0	LCK-3
aguggaagugccaugaacuugauccaga	1	0	JCK-3
guggaagugccaugaacuugaucc	4	0	JT-3
guggaagugccaugaacuugaucc	2	0	JCK-3
guggaagugccaugaacuugaucc	2	0	JT-2
guggaagugccaugaacuugaucc	1	0	JCK-1
guggaagugccaugaacuugauccaU	3	1	LCK-3
guggaagugccaugaacuugauccagU	1	1	JCK-2
guggaagugccaugaacuugauccagU	1	1	JCK-3
guggaagugccaugaacuugauccaga	17	0	LCK-2
guggaagugccaugaacuugauccagU	1	1	LCK-3
guggaagugccaugaacuugauccaga	1	0	LT-2
guggaagugccaugaacuugauccaga	1	0	JCK-3
guggaagugccaugaacuugauccagU	4	1	JCK-1
guggaagugccaugaacuugauccaga	4	0	JCK-2
guggaagugccaugaacuugauccaga	27	0	LCK-1
uggaaUugccaugaacuuga	1	1	JCK-3
uggaaUugccaugaacuuga	1	1	JCK-2
uggaaUugccaugaacuugau	1	1	JCK-1
uggaagugccaugaacuugau	2	0	LT-3
uggaaUugccaugaacuugau	3	1	JCK-3
uggaaUugccaugaacuugau	2	1	JCK-2
uggaagugccaugaacuugauU	1	1	JCK-2
uggaagugccaugaacuugauU	1	1	JCK-3
uggaagugccaugaacuugauc	4	0	JCK-3
uggaagugccaugaacuugauc	1	0	LT-2
uggaagugccaugaacuugauU	2	1	LT-3
uggaaUugccaugaacuugauc	3	1	JCK-1
uggaagugccaugaacuugauA	1	1	LT-1
uggaagugccaugaacuugauc	1	0	JCK-1
uggaagugccaugaacuugauc	2	0	JCK-2
uggaaUugccaugaacuugauc	7	1	JCK-2
uggaagugccaugaacuugaucc	3	0	LT-1
uggaaUugccaugaacuugaucc	2	1	JT-3
uggaagugccaugaacuugaucc	3	0	LT-3
uggaagugccaugaacuugaucc	2	0	LT-2
uggaaUugccaugaacuugaucc	2	1	JT-2
uggaaUugccaugaacuugaucc	4	1	JCK-1
uggaagugccaugaacuugaucc	3	0	JCK-1
uggaagugccaugaacuugaucc	7	0	JCK-2
uggaagugccaugaacuugaucc	2	0	JCK-3
uggaaUugccaugaacuugaucc	6	1	JCK-3
uggaagugccaugaacuugaucc	5	0	LCK-3
uggaaUugccaugaacuugaucc	21	1	JCK-2
uggaaUugccaugaacuugaucca	8	1	JCK-2
uggaaUugccaugaacuugaucca	8	1	JCK-3
uggaaUugccaugaacuugaucca	3	1	JCK-1
uggaagugccaugaacuugauccagU	1	1	LT-3
uggaagugccaugaacuugauccagC	2	1	LT-2
gaagugccaugaacuugauccaA	1	1	LT-1
aagugccaugaacuugUu	1	1	LT-1

Mature **Star**

uccaucuuguucguucacccucuggaucuuguucgauggcacucccccuuguauccaaucucucucuuucucucucucucucuuauggugcgaguggaagugccaugaacuugauccagaaggugcucucgaacaaguuacaccuacuuaauaucuacuuucuucuuucuccauuacuauuauuaaugaugguuucuuuuucugauucugauuuuuaauuuauuuauuuguuuuuguguuuguguuu

aagugccaugaacuugau	5	0	LT-3
aagugccaugaacuugau	7	0	LT-1
aagugccaugaacuuUau	4	1	LCK-3
aaUugccaugaacuugau	1	1	JT-2
aaUugccaugaacuugau	3	1	JT-3
aaUugccaugaacuugau	9	1	JCK-2
aaUugccaugaacuugau	3	1	JCK-1
aagugccaugaacuugUu	2	1	LT-3
aagugccaugaacuugaA	2	1	LT-3
aaUugccaugaacuugau	19	1	JT-1
aagugccaugaacuugau	3	0	LT-2
aaUugccaugaacuugau	2	1	JCK-3
aagugccaugaacuugau	5	0	JCK-3
aagugccaugaacuugau	2	0	JCK-1
aagugccaugaacuugau	2	0	JT-3
aagugccaugaacuugau	6	0	JCK-2
aagugccaugaacuugauU	1	1	JCK-2
aaUugccaugaacuugauc	28	1	JT-1
aagugccaugaacuugauU	2	1	JCK-1
aagugccaugaacuugauc	8	0	JT-3
aagugccaugaacuugauU	4	1	JCK-3
aagugccaugaacuugauc	22	0	JCK-1
aaUugccaugaacuugauc	15	1	JCK-2
aagugccaugaacuugauU	2	1	LT-2
aaUugccaugaacuugauc	6	1	JCK-1
aagugccaugaacuugauA	1	1	LT-3
aagugccaugaacuugauc	1	0	LT-1
aagugccaugaacuugauc	15	0	JCK-2
aagugccaugaacuugauU	6	1	LT-1
aagugccaugaacuugauU	5	1	LT-3
aaUugccaugaacuugauc	26	1	JT-3
aaUugccaugaacuugauc	12	1	JCK-3
aCgugccaugaacuugauc	1	1	LT-2
aagugccaugaacuugauc	8	0	LT-3
aaUugccaugaacuugauc	21	1	JT-2
aagugccaugaacuugauc	1	0	LT-2
aagugccaugaacuugauc	3	0	JT-2
aagugccaugaacuugauc	23	0	JCK-3
aagugccaugCacuugauc	1	1	JCK-1
aagugcAaugaacuugaucc	1	1	JCK-2
aaUugccaugaacuugaucc	41	1	JCK-2
aagugccaugaacuugaucc	64	0	LT-2
aCgugccaugaacuugaucc	1	1	JCK-3
aagugccaugaacuugaucc	89	0	LCK-3
aagugccaugaacuugaucc	51	0	LT-1
aagugccaugaacuugaucc	21	0	LCK-2
aagugccaugaacuugaucA	3	1	JCK-3
aaUugccaugaacuugaucc	20	1	JT-2
aagugccaugaacuugaucc	62	0	JCK-1
aagugcAaugaacuugaucc	1	1	JCK-3
aCgugccaugaacuugaucc	1	1	LT-2
aagugccaugaacuugaucU	1	1	JCK-1
aaUugccaugaacuugaucc	25	1	JCK-3
aagugccaugaCcuugaucc	1	1	JCK-2
aagugccaugaacuugaucU	1	1	LT-3
aagugccaugaacuugaucA	2	1	JCK-1
aaUugccaugaacuugaucc	26	1	JCK-1
aagugccaugaacuugaucc	17	0	LCK-1
aagugccaugCacuugaucc	1	1	LT-1
aagugccaugaacuugaucU	2	1	LT-1
aagugccaugaacuugaucc	105	0	JCK-3
aagugccaugaacuugaucU	3	1	JCK-3
aagugccaugGacuugaucc	1	1	LT-3
aagugccaugaacuugaucU	3	1	JCK-2
aNgugccaugaacuugaucc	1	1	JCK-3
aagugccaugaacuugaucU	3	1	LT-2
aaUugccaugaacuugaucc	21	1	JT-3
aagugccaugaacuugaucc	108	0	JCK-2
Cagugccaugaacuugaucc	1	1	LT-2
aagugccaugaacuugaucU	6	1	LCK-3

Mature uggaucuuguucgauggcacuc — **Star** gugccaugaacuugauccaga

uccaucuuguucguucacccucuggaucuuguucgauggcacuccccuuguauccaaucucucucuuucucucucucucucucuuauggugcgaguggaagugccaugaacuugauccagaaggugcucucgaacaaguuacaccuacuuaauaucuacuuucuucuuucuccauuacuauuauuaaugaugguuucuuuuuucugauucugauuuuuaauuuauuuauuuguuuuuuguguuuguguuu

Read	Count	Mismatch	Library
aagugccaugaacuugaucc	93	0	LT-3
aagugccaugaacuugauccU	2	1	JCK-2
aagugccaugaGcuugaucca	1	1	JCK-1
aagugccaugaacuugaucca	33	0	LT-1
aagugccaugaacuugaucca	33	0	LT-2
aaNugccaugaacuugaucca	1	1	LT-2
aaUugccaugaacuugaucca	14	1	JCK-3
aagugcAaugaacuugaucca	1	1	JCK-2
aagugccaugaacuugaucca	184	0	JCK-2
aaguAccaugaacuugaucca	1	1	LT-2
aagugccaugaacuugauccU	1	1	LT-1
aagugccaugaacuugaGcca	1	1	JCK-1
aagugccaugaacuugaucca	6	0	JT-3
Cagugccaugaacuugaucca	1	1	JCK-3
aagugUcaugaacuugaucca	1	1	JCK-3
aagugccaugaacuugauccC	2	1	LT-3
aagugccaugaacuugauccC	1	1	JCK-2
aCgugccaugaacuugaucca	1	1	LT-2
aagugccaugaacuugauccU	2	1	LT-3
aagugccaugaacuugaucca	41	0	LT-3
aagugccaugaacuugaucca	120	0	JCK-3
aagugccaugaacuugaucca	93	0	JCK-1
aagugccaugaacuugaucca	7	0	JT-2
aagugccaugaacuugauccC	2	1	JCK-3
aagugccCugaacuugaucca	1	1	JCK-3
aaUugccaugaacuugaucca	10	1	JCK-1
aagugccaugaacuugaucca	5	0	LCK-3
aaUugccaugaacuugaucca	19	1	JCK-2
aNgugccaugaacuugaucca	1	1	JT-2
aagugccaugaacuugauccag	88	0	JCK-2
aagugccaugaacuugauccag	71	0	JCK-3
aagugccaugaacuugauccaU	2	1	JCK-2
aagugccaugaacuugaGccag	1	1	JCK-1
aagugccaugaacuugauccaA	1	1	LT-3
Cagugccaugaacuugauccag	3	1	LT-1
aagugccaugaacuugauccaU	6	1	JCK-3
aagugccaugaacuugauccag	29	0	LT-3
aagugccaugaacuugauccag	47	0	JCK-1
aagugccaugaacuugauccaA	1	1	JCK-3
aagugccaugaacuugauccag	27	0	LT-1
aagugccaugaacuugauccaA	2	1	JCK-1
aagugccaugaacuugauccag	17	0	LCK-3
aagugccaugaacuugauccaU	3	1	JCK-1
aagugccaugaacuugauccag	2	0	LT-2
aagugccaugaacuugauccaA	2	1	JCK-2
aagugccaugaacuugauccagC	1	1	JCK-3
aagugccaugaacuugauccagC	1	1	JCK-2
aagugccaugaacuugauccaga	5	0	JCK-1
aagugccaugaacuugauccagC	1	1	LT-1
aagugccaugaacuugauccaga	2	0	LT-3
aagugccaugaacuugauccaCa	2	1	JCK-2
aagugccaugaacuugauccagU	1	1	JCK-2
aagugccaugaacuugauccaCa	3	1	JCK-3
aagugccaugaacuugauccagU	2	1	JCK-1
aagugccaugaacuugauccaga	11	0	JCK-2
aagugccaugaacuugauccaga	2	0	LT-1
aagugccaugaacuugauccagC	2	1	JCK-1
aagugccaugaacuugauccagU	1	1	JCK-3
aagugccaugaacuugauccagU	2	1	LT-1
aagugccaugaacuugauccaga	4	0	JCK-3
aagugccaugaacuugauccaCa	1	1	JCK-1
aagugccaugaacuugauccagaaggug	1	0	JCK-3
aagugccaugaacuugauccaAaaggug	1	1	JCK-1
agugccaugaacuugauA	1	1	LT-1
agugccaugaacuugauc	7	0	LT-1
agugccaugaacuugauU	1	1	JCK-3
agugccaugaacCugauc	1	1	JCK-2
agugccaugaacuNgauc	1	1	LCK-1
agugccaugaacuugauc	19	0	LCK-2
agugccaugaacuugauc	5	0	JCK-1

Mature   Star

uccaucuuguucguucacccucuggaucuuguucgauggcacuccccuuguauccaaucucucucuuucucucucucucucuuauggugcgaguggaagugccaugaacuugauccagaaggugcucucgaacaaguuacaccuacuuaauaucuacuuucuucuuucuccauuacuauuauuaaugaugguuucuuuuuucugauucugauuuuuaauuuauuuauuuguuuuuguguuuguguuu

agugccCugaacuugauc	1	1	LT-2
agugccaugaacuugauc	11	0	LCK-1
agugccaugaacuugauc	15	0	LT-2
agugccaugaacuugauc	14	0	LT-3
agugccaugaacuugauc	6	0	LCK-3
agugccaugaacuugauc	5	0	JCK-3
agugccaugaacuugauc	10	0	JCK-2
agugccaugaacuugauU	2	1	LT-3
agugccaugaacuugaucU	1	1	LT-1
agugccaugaacuugGucc	1	1	LT-2
agugccaugaacuugaucc	46	0	LT-3
agugccaugaacuugaucc	47	0	LCK-2
aUugccaugaacuugaucc	3	1	JCK-2
agugccaugaacuugaucc	22	0	JCK-3
agugccaugaacuugaucA	2	1	LT-2
agugccaugaacuugaucc	6	0	JT-3
agugccaugaacuugaucc	27	0	JCK-1
agugccaugaacuugaucc	45	0	LCK-3
agugccaugaacuugaucc	30	0	JCK-2
agugccaugaacuugaucc	19	0	LT-1
aUugccaugaacuugaucc	2	1	JCK-3
agugccaugaacuugaucc	51	0	LT-2
agugccaugaacuugaucc	3	0	JT-2
agugccaugaacuugaucc	41	0	LCK-1
agugccaugaacuugaucca	27	0	LT-2
agugccaugaacuugaucca	12	0	JT-3
agugccaugaacuugaucca	11	0	JCK-3
agugccaugaacuugCucca	1	1	LT-2
agugccaugaacuugaucca	10	0	JCK-1
aUugccaugaacuugaucca	2	1	JCK-2
agugccaugaacuugaucca	51	0	LCK-3
Cgugccaugaacuugaucca	1	1	LCK-3
agugccaugaacuugaucca	20	0	LCK-1
aguAccaugaacuugaucca	1	1	JCK-2
agugccaugaacuugaucca	25	0	JCK-2
agugccaugaacuugaucca	5	0	JT-2
aUugccaugaacuugaucca	1	1	JCK-3
agugccaugaacuugaucca	18	0	LT-3
agugccaugaacuugaucca	22	0	LCK-2
aUugccaugaacuugaucca	1	1	JCK-1
agugccaugaacuugaucca	20	0	LT-1
agugccaugaacuugauccag	23	0	LCK-3
agugccGugaacuugauccag	1	1	JCK-3
Cgugccaugaacuugauccag	1	1	JCK-3
agugccaugaacuugauccag	50	0	LT-1
agugccaugaacuugauccaU	4	1	JCK-3
aUugccaugaacuugauccag	2	1	JCK-3
agugccaugaacuugauccag	28	0	LT-2
agugccaugaacuugauccaU	2	1	LT-1
agugccaugaacuugauccCg	1	1	JCK-2
agugccaugaacuugaCccag	1	1	LT-3
aguAccaugaacuugauccag	1	1	LT-3
agugccaugaacuugauccaA	3	1	JCK-2
agugccaugaacuugauccaA	1	1	JCK-1
agugccaugaacuugauccaU	3	1	JCK-2
aUugccaugaacuugauccag	1	1	JCK-2
agugccaugaacuugauccaA	1	1	LT-1
agugccaugaacuugauccaA	1	1	JCK-3
agugccaugaacuugauccag	120	0	JCK-3
agugccaugaacuugauccag	58	0	LT-3
agugccaugaacuugauccag	179	0	JCK-2
agugccaugaacuugauccaU	4	1	JCK-1
agugccCugaacuugauccag	1	1	JCK-2
agugccaugaacuugauccag	115	0	JCK-1
Cgugccaugaacuugauccaga	4	1	JCK-1
Cgugccaugaacuugauccaga	2	1	JCK-3
Ugugccaugaacuugauccaga	1	1	LT-3
Cgugccaugaacuugauccaga	4	1	JCK-2
agugccaugaacuugauccagU	2	1	JCK-3
agugccaugaacuugauccagU	6	1	JCK-1

Mature | Star

uccaucuuguucguucacccucuggaucuuguucgauggcacuccccuuguauccaaucucucucuuucucucucucucucuuauggugcgaguggaagugccaugaacuugauccagaaggugcucucgaacaaguuacaccuacuuaauaucuacuuucuucuuucuccauuacuauuauuaaugaugguuucuuuuuucugauucugauuuuuaauuuauuuauuuguuuuuuguguuuguguuu

agugccaugaacuugauccaga	6	0	LT-3
agugccaugaacuugauccaAa	1	1	JCK-3
agugccaugaacuugauccaga	2	0	LT-2
agugccaugaacuugauccagC	1	1	JCK-2
agugccaugaacuugauccagU	4	1	JCK-2
Cgugccaugaacuugauccaga	1	1	LT-1
agugccaugaacuugauccaga	5	0	JCK-3
agugccaugaacuugauccaga	16	0	JCK-2
agugccaugaacuugauccaga	9	0	JCK-1
agugccaugaacuugauccaAa	1	1	JCK-2
agugccaugaacuugauccaga	2	0	LT-1
agugccaugaacuugauccagUa	1	1	LT-1
gugccaugaacuugaucc	26	0	JCK-1
Uugccaugaacuugaucc	3	1	JT-1
gugccaugaacuugaucA	5	1	JCK-3
gugccaugaacuugaucc	34	0	LT-1
Uugccaugaacuugaucc	32	1	JCK-2
gugccaugaacuugaucc	40	0	JCK-3
gugccaugaacuugaucc	40	0	LCK-2
gugccaugaacuugaucc	41	0	LCK-3
Augccaugaacuugaucc	1	1	LT-2
gugccaugaacuugaucU	1	1	LT-3
Augccaugaacuugaucc	2	1	LCK-1
gugccaugaacuugaucA	8	1	JCK-2
gugccaugaacuugaucc	34	0	LCK-1
gugccaugaacuugaucc	51	0	JCK-2
gugccaugaacuugaucc	11	0	JT-1
gugccaugaacuugaucc	69	0	LT-3
Uugccaugaacuugaucc	15	1	JCK-1
gugccaugaacuugaucc	35	0	LT-2
Uugccaugaacuugaucc	19	1	JCK-3
Uugccaugaacuugaucca	5	1	JCK-1
gugccaugaacuugaucca	6	0	JCK-1
Uugccaugaacuugaucca	3	1	JCK-2
gugccaugaacuugaucca	22	0	LT-3
gugccaugaacuugaucca	14	0	JT-1
gugccaugaacuugaucca	27	0	LT-2
gugccaugaacuugaucca	6	0	JCK-3
gugccaugaacuugaucca	11	0	JCK-2
gugccaugaacuugaucca	19	0	LCK-3
gugccaugaacuugaucca	14	0	LT-1
Uugccaugaacuugaucca	2	1	JCK-3
gugccaugaacuugauccaA	5	1	LT-3
gugccaugaacuugauccaA	3	1	JCK-1
gugccaugaCcuugauccag	1	1	JCK-2
gugccaugaacuugauccaU	1	1	JCK-2
gugccaugaacuugauccaA	3	1	LT-1
gugccaugaacuugauccaA	7	1	JT-3
Uugccaugaacuugauccag	79	1	JCK-2
gugccaugaCcuugauccag	1	1	JT-3
Uugccaugaacuugauccag	34	1	JCK-1
gugccaugaaAuugauccag	1	1	LT-2
Augccaugaacuugauccag	1	1	JCK-2
Uugccaugaacuugauccag	1	1	LT-2
Augccaugaacuugauccag	1	1	JT-3
gugccaugaacuugauccag	125	0	LT-3
gugccaugaacuugauccag	47	0	JT-1
gugccaugaacuugauccag	46	0	LCK-1
gugccaugaacuugauccag	26	0	JT-3
gugccaugaacuugauccaU	6	1	LT-2
gugccaugaacuugauccag	221	0	JCK-2
Uugccaugaacuugauccag	1	1	LT-3
gugccaugaacCugauccag	1	1	LT-2
gugccaugaacuugauccag	163	0	JCK-3
gugccaugaacuugauccag	138	0	JCK-1
gugccaugaacuugauccaA	4	1	JT-2
gugccaugaacuugCuccag	1	1	LT-3
Uugccaugaacuugauccag	10	1	JT-3
gugccaugaacuugauccag	16	0	JT-2
gugccaugaacuugauccaU	1	1	JCK-1

Mature **Star**

uccaucuuguucguucacccucuggaucuuguucgauggcacuccccuuguauccaaucucucucuuucucucucucucucuuauggugcgaguggaagugccaugaacuugauccagaaggugcucucgaacaaguuacaccuacuuaauaucuacuuucuucuuucuccauuacuauuauuaaugaugguuucuuuuuucugauucugauuuuuaauuuauuuauuuguuuuuuguguuuguguuu

gugccaugaCcuugauccag	1	1	LT-2
gugccaugaacuugauccaA	4	1	JCK-3
gugccaugaacuugauccag	103	0	LT-1
gugccaugaacuugauccag	63	0	LCK-3
Uugccaugaacuugauccag	20	1	JT-1
gugccaugaacuugauccag	56	0	LCK-2
gugccaugCacuugauccag	1	1	LT-1
Augccaugaacuugauccag	1	1	LCK-2
Uugccaugaacuugauccag	6	1	JT-2
gugccaugaacuugauccag	84	0	LT-2
gugccaugaacuugauccaU	2	1	JCK-3
gugccaugaacuugauccaA	8	1	JCK-2
Uugccaugaacuugauccag	66	1	JCK-3
gugccaugaacuugauccagC	1	1	JCK-2
Augccaugaacuugauccaga	7	1	JCK-3
gugccaugaacuugauccagU	8	1	JCK-2
gugccaugaacCugauccaga	1	1	JCK-1
gugccaugaacuugCuccaga	2	1	JCK-2
gugccaugaacuugauccagC	1	1	LT-2
gugccaugaacuugauccaUa	1	1	LT-3
gugccaugaacuugaucGaga	2	1	JCK-1
gugccaugaacuugauccaga	134	0	JT-1
gugccCugaacuugauccaga	2	1	LT-2
gugccaugCacuugauccaga	1	1	JCK-2
gugccaugaacuugauccaga	463	0	LT-2
Augccaugaacuugauccaga	3	1	JCK-1
gugccaugaacuugauccaNa	3	1	JCK-1
gugccCugaacuugauccaga	4	1	JCK-2
gugccCugaacuugauccaga	1	1	LT-3
gugccGugaacuugauccaga	1	1	JCK-1
gugccCugaacuugauccaga	4	1	JCK-1
gugccaugaacuugauccCga	1	1	LT-1
gugccaugaacuugauccaga	2129	0	JCK-3
guNccaugaacuugauccaga	1	1	LT-2
gugccaugaacuugauccagU	5	1	LT-1
gugccaugaacuugauccaga	295	0	LCK-3
gugccaugGacuugauccaga	1	1	JCK-1
gCgccaugaacuugauccaga	1	1	LT-3
Uugccaugaacuugauccaga	871	1	JCK-3
gugUcaugaacuugauccaga	2	1	JCK-2
gugccaugaacuugauccCga	1	1	LCK-1
gugccaugaacuugauccCga	2	1	JCK-1
gugccaugCacuugauccaga	2	1	LT-2
gugcAaugaacuugauccaga	2	1	JCK-1
Cugccaugaacuugauccaga	1	1	JCK-1
gNgccaugaacuugauccaga	1	1	JCK-3
gugccaugaacuugauccaga	90	0	LCK-1
gugccaugaacuugauccCga	1	1	LCK-3
Uugccaugaacuugauccaga	1	1	JT-1
gugccaugaacuugauccCga	1	1	JT-1
gugccaugaacuugCuccaga	1	1	JCK-1
gugccaugaacuCgauccaga	1	1	JCK-2
gugccaugaacuCgauccaga	1	1	JCK-3
gCgccaugaacuugauccaga	2	1	JCK-3
gugccaugaacuugauccagG	1	1	JCK-2
gugUcaugaacuugauccaga	2	1	JCK-3
gugccaugaacuugauccGga	1	1	JCK-3
gugccaugaacuugauccaUa	2	1	LT-1
Augccaugaacuugauccaga	3	1	LT-3
gNgccaugaacuugauccaga	3	1	JCK-2
gugccaugaacuugauccagU	16	1	LCK-1
gugccaugaacuugaucUaga	2	1	JCK-1
gugccaugaacNugauccaga	1	1	JT-1
gugccaugaacuugauccagU	9	1	LT-2
Augccaugaacuugauccaga	4	1	JCK-2
gugccaugaacuugauccagU	6	1	JCK-1
gugccCugaacuugauccaga	2	1	JCK-3
gugccaugaCcuugauccaga	2	1	JCK-1
gugccaugaacuugauccaga	56	0	JT-2
gugccaugaacuCgauccaga	1	1	JCK-1

Mature — **Star**

uccaucuuguucguucacccucuggaucuuguucgauggcacuccccuuguauccaaucucucucuuucucucucucucucuuauggugcgaguggaagugccaugaacuugauccagaaggugcucucgaacaaguuacaccuacuuaauaucuacuuucuucuuucuccauuacuauuauuaaugaugguuucuuuuuucugauucugauuuuuaauuuauuuauuuguuuuuuguguuuguguuu			
gugccaugaacuugauccagU	22	1	LT-3
gugccaugaacuugauccagU	10	1	JCK-3
gugccaugaCcuugauccaga	2	1	JCK-2
Uugccaugaacuugauccaga	4	1	JT-2
gugccaugaacuugauccaAa	4	1	JCK-2
gugccaugaacNugauccaga	7	1	JCK-1
gugccaugaacuugauccCga	1	1	LT-3
gugccaugNacuugauccaga	1	1	JCK-3
Cugccaugaacuugauccaga	4	1	JCK-2
gugccaugaacuugauccaga	2906	0	JCK-2
gugccNugaacuugauccaga	1	1	LCK-1
Cugccaugaacuugauccaga	4	1	JCK-3
gugccaugaacuugaucGaga	1	1	JCK-3
gNgccaugaacuugauccaga	2	1	JCK-1
gugcAaugaacuugauccaga	1	1	JT-1
Uugccaugaacuugauccaga	1141	1	JCK-2
gugccCugaacuugauccaga	2	1	LT-1
Cugccaugaacuugauccaga	1	1	LT-3
gugccaugaacuugauccaAa	5	1	LT-1
gugccaugaacuugauccagC	7	1	LT-3
gugccaugaacuuUauccaga	1	1	JCK-1
gugccaugaacuugauccaAa	2	1	LT-2
gugccCugaacuugauccaga	1	1	JT-1
gugccaugaacuugauccaga	107	0	LCK-2
gugcUaugaacuugauccaga	2	1	JCK-2
gugcUaugaacuugauccaga	3	1	JCK-3
gugccaugCacuugauccaga	1	1	JCK-3
gugccaugaacuugauccaga	1669	0	JCK-1
gugccaugaCcuugauccaga	1	1	LCK-3
gugccaugaacuugCuccaga	1	1	LT-1
gugccaugaacuugauccaAa	4	1	JCK-1
gugccaugaacuugauccagC	2	1	LT-1
gugccaugaacuugauccaga	649	0	LT-3
gugccaugaacuugauccaAa	1	1	LCK-3
gugccaugaacuugauccagU	9	1	JT-1
gugccaugaacuugauccaCa	1	1	JCK-1
gugccaugaacuugauccaga	43	0	JT-3
gugccaugaacuugauccaAa	7	1	LT-3
gugccaugaacuugauccaAa	9	1	JCK-3
Uugccaugaacuugauccaga	6	1	JT-3
guAccaugaacuugauccaga	2	1	JCK-2
Uugccaugaacuugauccaga	666	1	JCK-1
gugccaugaacuugauccagU	25	1	LCK-2
gugccaugaacuugCuccaga	2	1	LT-3
gugccaugaacuugauccaUa	1	1	JCK-2
gugccaugaacuugauccaga	444	0	LT-1
gugccaugaacuugauccNga	1	1	JCK-1
gugccaugaacuugCuccaga	1	1	LT-2
Augccaugaacuugauccaga	1	1	LT-1
guAccaugaacuugauccaga	1	1	JCK-3
Uugccaugaacuugauccaga	1	1	LCK-3
gugccaugaacuugauccagaU	3	1	JCK-2
gugccaugaacuugauccagaa	2	0	LT-1
gugccaugaacuugauccagaa	4	0	JCK-1
gugccaugaacuugauccagaU	3	1	JCK-1
gugccaugaacuugauccagaU	2	1	JCK-3
gugccaugaacuugauccagaC	4	1	LT-1
gugccaugaacuugauccagaC	4	1	LT-2
gugccaugaacuugauccagaU	1	1	LT-1
gugccaugaacuugauccagaC	2	1	JCK-2
gugccaugaacuugauccagaC	3	1	LT-3
Uugccaugaacuugauccagaa	2	1	JCK-2
gugccaugaacuugauccagaa	5	0	LT-3
gugccaugaacuugauccagaC	2	1	JCK-1
gugccaugaacuugauccagaa	9	0	JCK-3
gugccaugaacuugauccaAaa	1	1	JCK-2
gugccaugaacuugauccagaa	11	0	LCK-3
Uugccaugaacuugauccagaa	1	1	JCK-1
Uugccaugaacuugauccagaa	4	1	JCK-3
gugccaugaacuugauccagaa	8	0	JCK-2

Mature **Star**

uccaucuuguucguucacccucuggaucuuguucgauggcacucccuuguauccaaucucucucuuucucucucucucucuuauggugcgaguggaagugccaugaacuugauccagaaggugcucucgaacaaguuacaccuacuuaauaucuacuuucuucuuucuccauuacuauuauuaaugaugguuucuuuuuucugauucugauuuuuaauuuauuuauuuguuuuuuguguuuguguuu

gugccaugaacuugauccagaaA	8	1	JCK-2
gugccaugaacuugauccagaaA	3	1	JCK-3
gugccaugaacuugauccagaaA	3	1	JCK-1
gugccaugaacuugauccagaagguU	2	1	JCK-3
.ugccaugaacuugaucca	6	0	LT-3
.ugccaugaacuugaucca	1	0	JCK-3
.ugccaugaacuugaCcca	1	1	LT-3
.ugccaugaacuugaucca	7	0	JCK-2
.ugccaugaacuugaucca	1	0	JCK-1
.ugccaugaacuugaucca	8	0	LCK-3
.ugccaugaacuugaucca	1	0	LT-1
.ugccaugaacuugaucca	7	0	LT-2
.Cgccaugaacuugauccag	1	1	JCK-1
.ugccaugaacuugauccag	114	0	LT-2
.ugccaugaacuugauccaA	2	1	JCK-3
.uNccaugaacuugauccag	1	1	JCK-1
.ugccaugaacuugauccaA	1	1	JCK-2
.ugccauCaacuugauccag	1	1	JCK-1
.ugccaCgaacuugauccag	1	1	JCK-2
.ugccaugaacuugauccCg	1	1	JCK-2
.ugccaugaacuugauccag	43	0	JT-3
.ugccaugCacuugauccag	1	1	JCK-3
.ugccaugaacuugauccCg	1	1	LT-2
.ugccaugaCcuugauccag	1	1	LT-1
.ugccaugaacuugauccag	101	0	JCK-1
.ugccaugaacuugauccag	9	0	JT-1
.ugccaugaCcuugauccag	1	1	LT-2
.ugccaugaacuugauccaU	1	1	JCK-1
.ugccaugaacuugauccag	259	0	LT-3
.ugccaugaacuugauccag	102	0	JCK-3
.ugccaugCacuugauccag	1	1	LT-1
.ugccaugaacuugauccaA	1	1	JCK-1
.Cgccaugaacuugauccag	1	1	JCK-2
.ugccaugaacuugauccag	161	0	LT-1
.ugccaugaacuugauccag	74	0	LCK-3
.ugccaugaacuugauccaU	1	1	LT-3
.ugccaugaacuugauccag	26	0	JT-2
.Cgccaugaacuugauccag	2	1	JCK-3
.ugccaugaacuugauccag	167	0	JCK-2
.ugccaugaCcuugauccag	1	1	LT-3
.ugccaugaacuugauccagN	1	1	JCK-1
.ugccaugaacuugauccaga	6	0	JT-2
.ugccaugCacuugauccaga	1	1	LCK-3
.ugccaugaacuugauccaAa	1	1	JCK-3
.ugccaugaacuugauccagU	7	1	JCK-3
.ugccGugaacuugauccaga	1	1	LT-1
.ugccaugaacuugCuccaga	1	1	LCK-3
.ugccaugaCcuugauccaga	4	1	JCK-3
.ugccaugaacuugauccagG	1	1	JCK-2
.ugccaugaacuugauccaga	184	0	LCK-3
.ugccaugaacuugauccaga	86	0	LCK-2
.ugccCugaacuugauccaga	1	1	LT-1
.ugccaugaacuugauccagU	5	1	JCK-2
.ugccaugCacuugauccaga	1	1	JT-1
.uUccaugaacuugauccaga	1	1	JCK-3
.ugccaugaacuugauccaga	290	0	LT-2
.ugccaugaacuugauccagU	1	1	JCK-1
.ugccaugaacuugauccagC	3	1	JCK-2
.ugccaugaacuugauccagG	1	1	LT-2
.ugNcaugaacuugauccaga	1	1	LT-2
.ugccaugaacuCgauccaga	1	1	JCK-3
.ugccaugaCcuugauccaga	1	1	JCK-1
.ugccaugCacuugauccaga	1	1	LT-3
.uNccaugaacuugauccaga	2	1	JCK-1
.ugccaugaacuugauccaga	848	0	JCK-1
.ugcAaugaacuugauccaga	1	1	JCK-2
.ugccaugaacuugCuccaga	1	1	LT-3
.ugcGaugaacuugauccaga	2	1	JCK-1
.uAccaugaacuugauccaga	1	1	LCK-3
.ugccaugaacuugauccCga	3	1	JCK-2

Mature — Star

uccaucuuguucguucacccucuggaucuuguucgauggcacuccccuuguauccaaucucucucuuucucucucucucucucuuauggugcgaguggaagugccaugaacuugauccagaaggugcucucgaacaaguuacaccuacuuaauaucuacuuucuucuuucuccauuacuauuauuaaugaugguuucuuuuuucugauucugauuuuuaauuuauuuauuuguuuuuuguguuuguguuu

ugccaugaacuugauccagG	1	1	JCK-1
ugccaugaacuugaCccaga	1	1	JCK-3
ugccaugaacuugCuccaga	1	1	JT-1
Cgccaugaacuugauccaga	1	1	LCK-3
ugccaugaacuugauccagU	3	1	LT-1
ugccaugaacuugauccagU	4	1	LT-3
ugccaugaacuugauccaNa	1	1	JCK-1
ugccaugaCcuugauccaga	1	1	LT-1
ugccaugaacuugauccagC	4	1	LT-3
ugccCugaacuugauccaga	1	1	JCK-2
ugccaugaacuugauccaga	1507	0	JCK-2
ugccaugaacuugauccCga	2	1	JCK-1
ugccaugGacuugauccaga	1	1	JCK-2
uNccaugaacuugauccaga	2	1	JCK-3
ugccaugaacuNgauccaga	1	1	JCK-1
ugccaugaacuugauccaga	93	0	JT-1
uAccaugaacuugauccaga	1	1	JCK-2
ugccaugCacuugauccaga	1	1	LT-2
ugcAaugaacuugauccaga	1	1	JCK-1
ugccaugaacuugauNcaga	1	1	JCK-3
ugccaugaacuugauccaga	12	0	JT-3
ugccaugaacuugauccaga	476	0	LT-1
ugccauAaacuugauccaga	1	1	LT-3
ugccCugaacuugauccaga	1	1	LT-3
ugccaugaCcuugauccaga	1	1	JCK-2
ugccaugaacuugauccaga	657	0	LT-3
uAccaugaacuugauccaga	2	1	LT-1
ugccaugaacuuUauccaga	1	1	JCK-2
ugccCugaacuugauccaga	4	1	JCK-3
ugccaugaacuugauccagC	2	1	JCK-3
ugccCugaacuugauccaga	1	1	LT-2
ugccaugaacuugauccagU	9	1	LT-2
ugccaugaacuugauccaga	60	0	LCK-1
ugccaugaacuugauccaga	1134	0	JCK-3
ugccaugaacuuUauccaga	1	1	JCK-3
ugccaugaacuugauccagaU	9	1	LT-3
ugccaugaacuugauccagaa	4	0	LT-2
ugccaugaacuugauccagaU	5	1	LT-2
ugccaugaacuugauccagaU	2	1	JT-3
ugccaugaacuugauccagaC	3	1	LT-1
ugccaugaacuugauccagaU	10	1	LT-1
ugccaugaacuugauccagaa	3	0	JCK-3
ugccaugaacuugauccagaU	1	1	JCK-1
ugccaugaacuugauccagaU	2	1	JCK-2
ugccaugaacuugauccagaa	5	0	JCK-1
ugccaugaacuugauccagaC	5	1	LT-3
ugccaugaacuugauccagaa	1	0	JCK-2
ugccaugaacuugauccagaU	5	1	LCK-3
ugccaugaacuugauccagaag	2	0	JCK-3
ugccaugaacuugauccagaag	1	0	JCK-2
ugccaugaacuugauccagaagg	5	0	LT-3
ugccaugaacuugauccagaagg	3	0	JCK-3
ugccaugaacuugauccagaagg	1	0	JCK-2
ugccaugaacuugauccagaagg	1	0	JCK-1
gccaugaacuugauccag	1	0	JCK-2
gccaugaacuugauccaga	1	0	JCK-3
gccaugaacuugauccaga	1	0	JCK-1
ccaugaacuugauccagU	1	1	JCK-3
ccaugaacuugauccagU	4	1	LCK-3
ccaugaacuugauccaga	1	0	JCK-2
ccaugaacuugauccagU	2	1	JCK-1
ccaugaacuugauccaga	2	0	LT-3
ccaugaacuugauccagaaggu	1	0	LT-3
aacuugauccagaaggugcucu	1	0	JCK-1
aacuugauccagaaggugcucu	1	0	JCK-2
acuugauccagaaggugcucucga	1	0	LT-2
uccagaaggugcucucgaacaaguuac	1	0	LT-2
cagaaggugcucucgaacaagu	4	0	JCK-3
cagaaggugcucucgaacaagu	4	0	JCK-2
cagaaggugcucucgaacaagu	2	0	JCK-1

```
                Mature                                                                                          Star
uccaucuuguucguucacccucuggaucuuguucgauggcacuccccuuguauccaaucucucucuuucucucucucucucuuauggugcgaguggaagugccaugaacuugauccagaaggugcucucgaacaaguuacaccuacuuaauaucuacuuucuucuuucuccauuacuauuauuaaugaugguuucuuuuuucugauucugauuuuuaauuuauuuauuuguuuuuuguguuuguguuu
...................................................................................................................cagaaggugcucucgaacaagu...............................................................................................................	3	0	LT-3
...................................................................................................................cagaaggugcucucgaacaagu...............................................................................................................	7	0	LCK-3
...................................................................................................................cagaaggugcucucgaacaagu...............................................................................................................	2	0	LT-1
....................................................................................................................agaaggugcucucgaacaagu...............................................................................................................	1	0	LT-1
....................................................................................................................agaaggugcucucgaacaagu...............................................................................................................	7	0	LT-3
....................................................................................................................agaaggugcucucgaacaagu...............................................................................................................	1	0	JCK-3
....................................................................................................................agaaggugcucucgaacaagu...............................................................................................................	3	0	JCK-2
....................................................................................................................agaaggugcucucgaacaagu...............................................................................................................	6	0	LCK-3
....................................................................................................................agaaggugcucucgaacaNgu...............................................................................................................	1	1	JCK-1
....................................................................................................................agaaggugcucucgaacaaguu..............................................................................................................	3	0	JCK-1
....................................................................................................................agaaggugcucucgaacaaguu..............................................................................................................	2	0	LT-3
....................................................................................................................agaaggugcucucgaacaaguu..............................................................................................................	1	0	LT-1
....................................................................................................................agaaggugcucucgaacaaguu..............................................................................................................	1	0	JCK-3
....................................................................................................................agaaggugcucucgaacaaguuaca...........................................................................................................	1	0	LT-3
.....................................................................................................................gaaggugcucucgaacaaguua.............................................................................................................	3	0	LT-1
.....................................................................................................................gaaggugcucucgaacaaguuac............................................................................................................	1	0	JCK-2
.....................................................................................................................gaaggugcucucgaacaaguuac............................................................................................................	2	0	JCK-3
.......................................................................................................................aggugcucucgaacaagu...............................................................................................................	1	0	LT-3
.......................................................................................................................aggugcucucgaacaaguA..............................................................................................................	1	1	LT-1
.......................................................................................................................aggugcucucgaacaaguuacC...........................................................................................................	1	1	LT-3
.......................................................................................................................aggugcucucgaacaaguuaca...........................................................................................................	1	0	LT-2
...................................................................................................................................................................................................uuuuuCcugauucugauu...................................	3	1	LT-3
...................................................................................................................................................................................................uuuuuuUugauucugauu...................................	2	1	LT-3
.........................................................................................................................................................................................................cugauucugauuuuuaau.............................	1	0	LT-2
```
